# Geometric morphometrics analysis of the hind wing of leaf beetles: proximal and distal parts are separate modules

**DOI:** 10.3897/zookeys.685.13084

**Published:** 2017-07-20

**Authors:** Jing Ren, Ming Bai, Xing-Ke Yang, Run-Zhi Zhang, Si-Qin Ge

**Affiliations:** 1 Key Laboratory of Zoological Systematics and Evolution, Institute of Zoology Chinese Academy of Sciences, Beijing, China; 2 University of Chinese Academy of Sciences, Beijing, China

**Keywords:** Chrysomelinae, evolution, variance, venation, wing folding

## Abstract

The success of beetles is mainly attributed to the possibility to hide the hindwings under the sclerotised elytra. The acquisition of the transverse folding function of the hind wing is an important event in the evolutionary history of beetles. In this study, the morphological and functional variances in the hind wings of 94 leaf beetle species (Coleoptera: Chrysomelinae) is explored using geometric morphometrics based on 36 landmarks. Principal component analysis and Canonical variate analysis indicate that changes of apical area, anal area, and middle area are three useful phylogenetic features at a subtribe level of leaf beetles. Variances of the apical area are the most obvious, which strongly influence the entire venation variance. Partial least squares analysis indicates that the proximal and distal parts of hind wings are weakly associated. Modularity tests confirm that the proximal and distal compartments of hind wings are separate modules. It is deduced that for leaf beetles, or even other beetles, the hind wing possibly exhibits significant functional divergences that occurred during the evolution of transverse folding that resulted in the proximal and distal compartments of hind wings evolving into separate functional modules.

## Introduction

Coleoptera (known as beetles) are the largest insect order, containing 380,000 named living species classified into more than 160 families (Mckenna et al. 2015); their success is partly attributed to the evolution of a tight exoskeletal shell that leaves no membranous areas exposed (Beutel and Haas 2000, Haas 2006). The fore wings of most beetles are hardened elytra which are not used for flight or to a very minor degree (in Archostemata), but mainly serve to form a protective cover for the hind part of the body (hind wings and abdomen) (Frantsevich 2011). As a part of this essential character complex of Coleoptera, a complicated hind wing folding mechanism has evolved (Beutel and Haas 2000, Haas 2006). As the flight organ, the hind wing must have a certain size to be aerodynamically functional, which makes them distinctly larger than the thickened fore wings (Haas et al. 2000).

Given that large and thin hind wings are vulnerable to damage, they must be folded not only longitudinally but also transversely to be stored below the elytra for protection during ground locomotion and especially when entering narrow spaces. The hind wings unfold only when needed, such as before flying (Muhammad et al. 2010, Truong et al. 2014). With flexed and folded wings, it is easier to hide, use small crevices and shelters against the impact of weather (e.g., wind and rain), and escape predators (Haas 2006). The fitness advantage is so great that transverse wing folding evolved in multiple insect orders (Coleoptera, Dermaptera, and some species of Blattodea) (Forbes 1926, Haas and Beutel 2001, Haas 2006, Beutel et al. 2014). In beetles, longitudinal folding was already present in the earliest stem-lineage representatives of the Lower Permian, whereas transverse folding evolved in the Middle Permian with the formation of a closed subelytral space (Beutel and Haas 2000).

**Figure 1.**
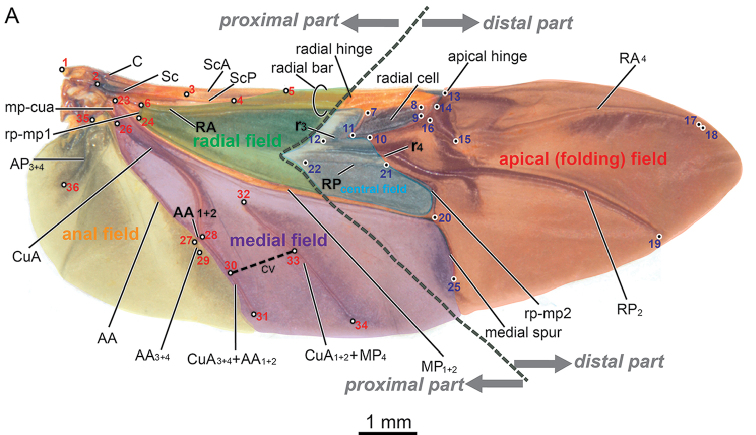
Leaf beetle hind wing (*Chrysomela populi* Linnaeus), with landmark locations (the dot with number), vein nomenclature and regional division. The nomenclature of the wing venation follows that of Kukalová-Peck & Lawrence (1993, 2004). Radial area: green, central area: blue, medial area: purple, anal area: yellow, apical (folding) area: red. Proximal part landmarks 1–6, 23, 24, and 26–36 mainly include radial, medial, and anal areas; distal part landmarks 7–22 and 25 include the central area, radial cell, and apical area. Abbreviations: Costa (C), Subcosta (Sc), Subcosta Anterior (ScA), Subcosta Posterior (ScP), Radius Anterior (RA), Radius Posterior (RP), Radial cross veins (r3, r4), Media Posterior (MP), Radio-medial cross veins (rp-mp1, rp-mp2), medial cross vein (cv), Cubitus Anterior (CuA), Medio-cubital Cross-vein or Arculus (mp-cua), Anal Anterior (AA), Anal posterior (AP). “+” indicates fused veins. The sub-number of veins reflects vein branches.

**Figure 2.**
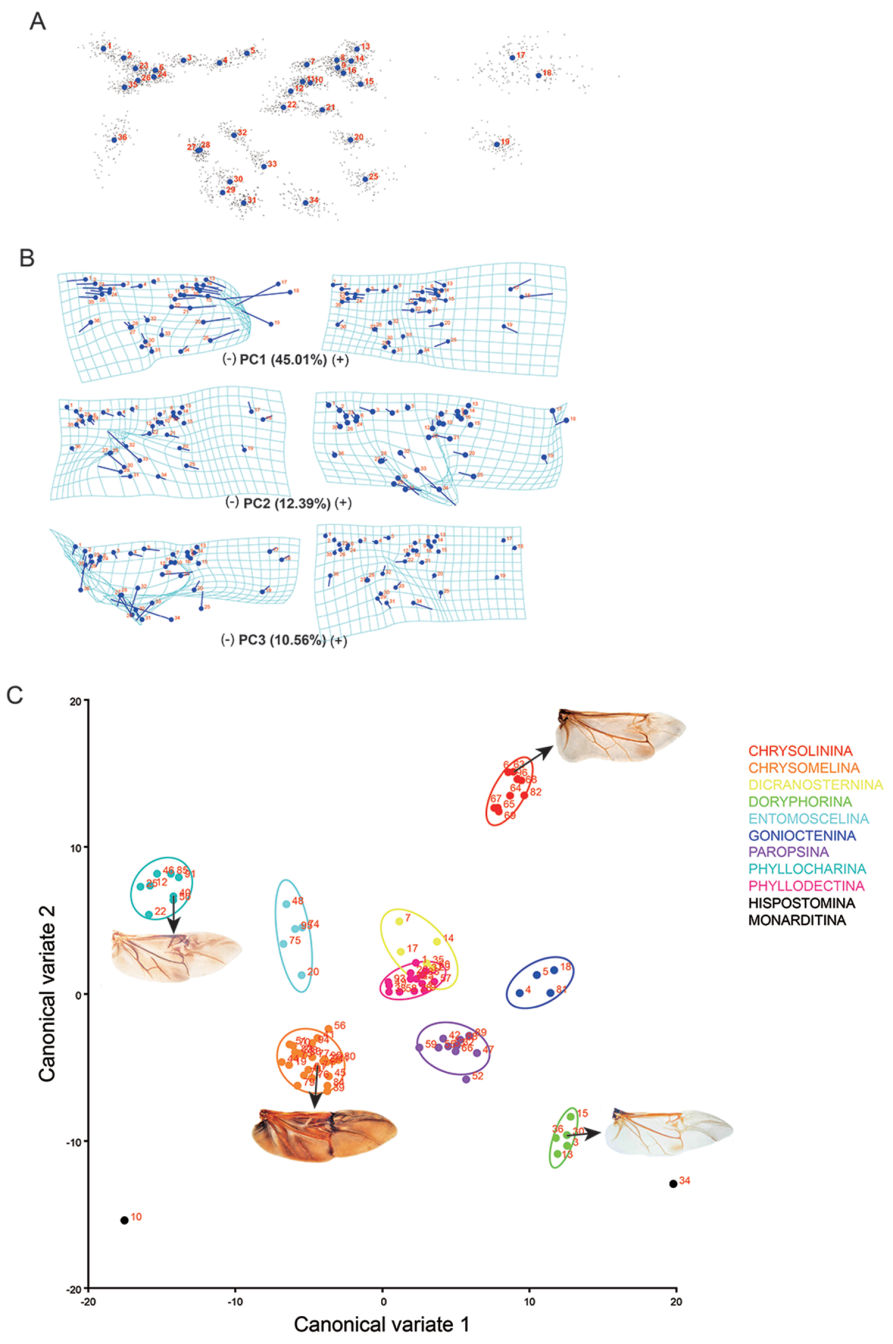
PCA and CVA results. **A** Centroid size graph of hind wing landmarks (Procrustes fit) **B** PCA results, the shape changes associated with the first three PCs: the relative size of the apical area which could be considered the main feature (variance contribution ratio was 45.01%) to influence of the overall variance of the hind wing, the changes of cross vein cv in the middle area (variance contribution ratio was 12.39%), and relative size of the anal area size (variance contribution ratio was 10.56%) **C** CVA results, the axis of CV1 and CV2 presented the first two large shape variance of all variance; points with different colours indicated different subtribes’ specimens; the ellipse is presented as an equal-frequency ellipse with a given probability level of 90%, which contains approximately 90% of the data points.

The apical area of beetle’s hind wings is folded under elytra when not flying. Transverse folding leads to some morphological changes of hind wings, for example, the wing size, but what additional changes are concomitant? In this study, the morphological variances of beetle hind wings were investigated using geometric morphometrics analysis based on Chrysomelinae beetles. Chrysomelinae beetles could be divided into two tribes, one is Timarchini which are not able to fly, hind wings are reduced or disappeared; the other is Chrysomelini which have functional hind wings (Seeno and Wilcox 1982).Since this study focuses on the wing variance, 96 specimens of tribe Chrysomelini (94 species, 81 genera, eleven subtribes) was collected to observe and analysis the hind wing variance. The typical hind wing of leaf beetles presented in the Figure 1. The hind wing was oblong and venation was simple. Usually, there were two main veins (R and M) and two cells (radial cell and cubitus-anal cell). Apical area is membrane. For some leaf beetles, there was a cross vein cv in the middle area of hind wings (Figure 2C). The wing variance was analysed based on the subtribe level. More importantly, the variation of wing venation caused by the transverse folding function was addressed based on geometric morphometrics analysis results.

Geometric morphometrics analysis approaches have been used successfully in evolutionary biology and systematics (Adams and Funk 1997, Klingenberg and McIntyre 1998, Klingenberg and Zaklan 2000, Shipunov and Bateman 2005, Herrel and O’Reilly 2006, Villemant et al. 2007, Wappler et al. 2012, Catalano et al. 2015, Outomuro and Johansson 2015, Werneburg et al. 2015). Mitteroecker and Gunz (2009) summarised the advances in geometric morphometrics. The geometric morphometric revolution has added to the sophistication of quantitative biological shape analysis while simultaneously simplifying data collection and analysis to answer phenotypic questions, including those related to shape (Mitteroecker and Gunz 2009, Lawing and Polly 2010). Using geometric morphometrics analysis, studies of the morphological variances of insect wings are the most commonplace (Klingenberg and Zaklan 2000, Villemant et al. 2007, Bai et al. 2011, Bai et al. 2012, Wappler et al. 2012, Krosch et al. 2013, Lorenz and Suesdek 2013, McCane 2013, Outomuro et al. 2013a, b). Most of these studies have focused on phylogenetic or taxonomic problems. Examples of geometric morphometrics applied to the morphology-function of wings are few. In this investigation, using geometric morphometrics analysis, Chrysomelinae beetle hind wings were investigated to explore their functional and morphological variances, especially for the apical area.

## Materials and methods

### Samples

This study was based on hind wing images (see Suppl. material 1) of 96 specimens (eleven subtribes, 81 genera, 94 species) of Chrysomelinae (Coleoptera, Chrysomelidae) to obtain landmark data. There were 94 species, 81 genera included in this study; each genus included one to three species. There was two species with two specimens from different geographical locations. There were 96 specimens in 94 species. The leaf beetle specimens were deposited in the Institute of Zoology, Chinese Academy of Sciences. The specimens were examined and dissected to obtain hind wings using a LEICA MZ 12.5 dissecting microscope. Pictures of the wings were obtained with a D500s Nikon camera connected to a stereoscope (Zeiss Stereo Discovery V12). The collection and subtribe information of 96 leaf beetle specimens is presented in Suppl. material 2.

### Landmark digitising

The tpsUtil (Version 1.44, Copyright 2009, F. James Rohlf, Ecology & Evolution, SUNY at Stony Brook) and tpsDig (Version 2.12, Copyright 2008 F. James Rohlf, Ecology & Evolution, SUNY at Stony Brook) software programs were used for digitising landmarks. Figure 1 illustrates the nomenclature of hind wing venation in chrysomelid and the location of 36 landmarks selected for the analysis. The locations of landmarks were chosen based on the intersection of veins or vein base, vein end or apex. The “anterior”, “posterior”, “proximal”, and “distal” veins or plates were used to describe the detailed position of landmarks. If a landmark did not exist on a certain wing, this landmark overlapped with next existing landmark. For example, specimen 70 (*Chrysomela populi* Linnaeus, Figure 1), landmark 30 and 33 didn’t not exist, then the landmark 30 overlapped with the next existing landmark 31, landmark 33 overlapped with landmark 34. The position description of landmarks is presented in Table 1. The landmark coordinate data are presented in Suppl. material 3. The nomenclature of the wing venation followed that of Kukalová-Peck and Lawrence (1993, 2004).

**Table 1.** Landmark position description.

Landmark #	Position Description
1	Proximal anterior point of humeral plate (HP)
2	The crossing point of BSc and Sc
3	The point of Sc getting to bifurcate into ScA and ScP
4	The crossing point of ScP and RA
5	The crossing point of ScA and RA
6	The crossing point of rp-m1 and RA
7	Proximal anterior point of radial cell
8	Distal anterior point of radial cell
9	Distal posterior point of radial cell
10	Anterior point of r4 (or the crossing point of r4 and radial cell)
11	Proximal posterior point of radial cell
12	Proximal point of r3
13	Apical hinge
14	The anterior point of triangular area of radial cell’s distal side
15	The posterior point of triangular area of radial cell’s distal side
16	The proximal point of triangular area of radial cell’s distal side
17	The distal point of RA_4_
18	The distal point of RA_1_
19	The distal point of RP_2_
20	The point of MP_1+2_ getting to bifurcate
21	The posterior point of r4, or the crossing point of r4 and rp-mp2
22	The proximal point of RP
23	Anterior point of mp-cua
24	The crossing point of rm-mp1and MP
25	The posterior of medial spur
26	Posterior point of mp-cua
27	The point of AA getting to bifurcate
28	The point of AA_1+2_ getting to fuse with CuA_3+4_
29	The posterior or distal point of AA_3+4_
30	The proximal point of cv
31	The posterior or distal point of AA_1+2_+CuA_3+4_
32	Anterior point of CuA_1+2_+MP_4_
33	The distal point of cv
34	Posterior point of CuA_1+2_+MP_4_
35	The base point of AP_3+4_
36	The posterior point of AP_3+4_

### Data analysis

MorphoJ (Version 1.06d) was used for landmark data analyses. MorphoJ is a software package enabling geometric morphometric analysis for two- and three-dimensional landmark data and designed for the analysis of actual biological data (Klingenberg 2011). Prior to further analyses, the landmark data (Suppl. material 3) of wings were imported into MorphoJ, and a complete Procrustes fit was conducted by orthogonal projection to correct size and orientation.

Principal component analysis (PCA): PCA is one of the most widely used methods for exploratory multivariate analysis (Klingenberg and McIntyre 1998, Klingenberg and Zaklan 2000). In this study, MorphoJ generates covariance matrices from landmark datasets of 96 specimens after Procrustes superimposition. Based on the covariance matrices, PCA was used to analyse and display the patterns of covariation of positions of landmarks throughout the wing. Principal components (PCs) are visualised directly as patterns of simultaneous displacements of landmarks in relation to one another.

Canonical variate analysis (CVA): CVA is a method used to find the shape features that best distinguish among multiple groups of specimens (Gumiel et al. 2003, Villemant et al. 2007). Group membership is a known priori. In this study, the subtribe was set as the known *a proiri* to test the phylogenetic implications of the wing features from PCV results. In the current data, there were 89 specimens (76 genera, 88 species) could be definitely divided into eleven subtribes; the left seven specimens (five genera, six species) had no clear subtribe information (Suppl. material 2). CVA based on the 89 specimens was used to explore the wing variance on subtribe level of leaf beetles.

Partial least squares (PLS) analysis: MorphoJ offers an implementation of PLS analysis between blocks of landmarks within the same configuration. This analysis identifies the features of shape variation that most strongly co-vary between the blocks and indicates their relative contribution to the total covariation between blocks (Rohlf and Corti 2000, Klingenberg et al. 2003, Klingenberg 2009). The hind wing was divided into two parts based on the transverse folding function of 96 beetles: landmarks 1–6, 23–24 and 26–36 served as the proximal part; landmarks 7–22 and 25 relevant to apical transverse folding served as the distal part. There were 10,000 permutation test rounds. The *RV* coefficient was used as a measure of overall covariation between blocks; this coefficient is a multivariate analogue of the squared correlation coefficient between two variables. When the *RV* coefficient values (between 0 and 1) are lower (< 0.5), the covariance of the two blocks is weak. The *RV* coefficient provided by this procedure is the same as that in the output from tests of modularity hypotheses (Escoufier 1973; Klingenberg 2009).

Modularity test: MorphoJ implements a method to evaluate hypotheses of modularity (Klingenberg 2009). Modularity is an important principle of organisation in biological systems that is also manifested at the morphological level (Klingenberg 2008). The hypothesis of independent variation in the proximal and distal wing parts as two modules (in terms of PLS analysis) was evaluated. MorphoJ can compare the strength of covariation between two partitions and either all or a large number of the possible alternative partitions with the same numbers of landmarks as in the hypothesised modules (Klingenberg 2009). Similar to PLS analysis, the *RV* coefficient is used as a measure of overall covariation between modules. If the hypothesis of modularity holds, the *RV* coefficient for the partition according to the hypothesis should be the lowest value, or it should at least be close the lower extreme of the distribution of *RV* coefficients for all of the partitions (Klingenberg 2009). Here, based on the two blocks of PLS, the configuration of 36 landmarks was partitioned into two subsets: one subset with 19 landmarks (1–6, 23–24, 26–36) and the other with 17 landmarks (7–22, 25). In our case, the total number of different partitions into subsets was approximately 9×10^9^ (Klingenberg 2009). However, landmark configurations with more than 20 landmarks may not be computationally feasible (Klingenberg 2009). Therefore, the random partitions of the configuration into subsets of the appropriate number of landmarks can be used instead. The recommended number of random partitions is in the order of 10^4^ for the comparison, which should provide a reasonable characterisation of the distribution of the *RV* coefficient (Klingenberg 2009). In this study, random partitions in the order of 10,000 and 1,000,000 were implemented, with contiguous partitions only.

## Results

### PCA and CVA results

In geometric morphometrics, allometry is widely characterised by multivariate regression of shape on size (usually centroid size or log-transformed centroid size); such regressions often fit the data well and the allometric shape changes tend to affect the entire structures (Klingenberg and Marugán-Lobón 2013). The Procrustes Fit was used to correct the size and orientation of wings. The Procrustes fit procedure adds a data matrix with the Centroid size to the dataset (Figure 2A). Based on Figure 2A, the 36 landmarks were reasonable to analysis the wing variance.

Based on the Procrustes fit data, PCA was carried out based on 96 specimens. The accumulative contribution ratio of the first three components was 68.04%, potentially indicating that the first three components represented the main shape variation of wing venation. Figure 2B shows that the first three PC shape changes. PC1 (with a variance contribution ratio of 45.01%) primarily affected the size of apical area of hind wing. PC1 (-) shows that landmarks 7–16, 20–22, and 25 moved distally, whereas landmarks 17–19 moved proximally, producing a smaller and shorter apical part. In addition, landmarks 1–4, 6, 23–24, 26, and 35 moved proximally, which made the radial and medial area smaller and shorter. Altogether, these changes produced a relatively smaller and shorter apical area of the hind wing. Similarly, PC1 (+) exhibited a relative larger and longer apical area of the hind wing. Therefore, the PC1 is better described as the variation of the relative size of the apical area of the hind wing between a larger and longer or a smaller and shorter apical area. PC2 (with a variance contribution ratio of 12.39%) primarily affected the size of the medial area of the hind wing by movements of landmarks 30 and 33 towards or away between landmarks 28, 32 and 31, 34, which presented the relative location and length of the cross vein cv or whether existed. In addition, landmark 29 exhibited a long variance distance, indicating the length of AA_3+4_. PC3 (with a variance contribution ratio of 10.56%) primarily affected the size of the anal area by the movement of landmarks 20, 22, 25, 29–34 and 36. To different degrees, both PC2 and PC3 affected the relative size of the apical area by moving proximal part landmarks 1–6, 24, 26, and 35 and distal part landmarks 17–19. Thus, when PC1, 2, and 3 are integrated, it was concluded that there were three features of the wing variance of leaf beetles: the relative size of the apical area which could be considered the main feature (variance contribution ratio of 45.01%) to influence of the overall variance of the hind wing, the changes of cross vein cv in the middle area, and relative size of the anal area size.

Based on the PCA wing variance results, CVA was used to test whether the three features were useful on the distinguishing of the subtribe level. There were eleven subtribes (89 specimens) of Chrysomelinae involved in this study, except subtribe and have one specimen for each (sample size is too small, there were no statistical significance); the left have 4–26 specimens for each subtribes. Iin CVA, there were nine subtribes with enough samples to do analysis. As the Figure 2C showed, the axis of CV1 and CV2 presented the first two large shape variance of all variance; points with different colour indicated different subtribes’ specimens; the ellipse is presented as an equal-frequency ellipse with a given probability level of 90%, which contains approximately 90% of the data points. The CVA result showed, in the nine subtribes, and couldn’t be divided based on wing shape data; these two subtribes were overlapped. The other seven subtribes could be divided clearly. Especially for subtribes distributing in the edge of coordinates (with big Mahalanobis distances each other): , Chrysomelina, Doryphorina, and , the four subtribes showed big large variances on wing shape. The wing difference between each other could be described as qualitative features. The typical hind wing images of the four subtribes were presented as the Figure 2C showing. beetles have a shorter apical area and cross vein cv in the middle area; while Chrysomelina beetles have longer apical area and no cross vein cv. Doryphorina beetles have a wide anal area and no cross vein cv; while have a narrow anal area and cross vein cv in the middle area. Doryphorina and mainly distributed along the axis of CV1 which had a big variance contribution ratio 40.19%; and Chrysomelina speciemns mainly distributed along the axis of CV2 which had a second big variance contribution ratio 27.83%. It was concluded that the relative size of the apical area and anal area, and cross vein cv in the middles area, could play an important role in the division of these 4 subtribes. For subtribes Entomoscelina, Gonioctenina, and , the wing variance also focused on the apical area, anal area and cross vein cv. These three subtribes distributed in the central region of coordinates (Mahalanobis distances are relatively small between each other); the difference between each other is quantitative and is hard to describe as qualitative features.

### PLS analysis and modularity test of the proximal and distal part of the hind wing

The apical area of the hind wings of beetles can be folded transversely under elytra (Forbes 1926). Both PCA and CVA showed that the apical area of wings has a large variance. These results prompted us to test whether the apical area had a relatively independent shape change in hind wing shape variances. The apical and central area and radical cells of hind wings (Figure 1) are involved in transverse folding in beetles. The landmarks involving transverse folding were selected as a block; all of the other landmarks composed a second block. PLS analysis of covariation within a configuration of landmarks 1–6, 23–24 and 26–36 as the proximal part and landmarks relevant to transverse folding 7–22 and 25 as the distal part (Figure 1) was performed to test the null hypothesis: no independent shape changes between the proximal and distal parts of the hind wing.

**Figure 3.**
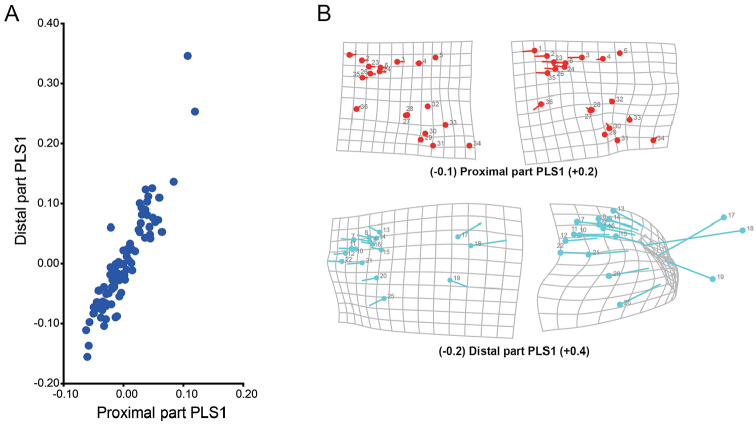
PLS analysis results. **A** Scatter plot of the PLS1 of two blocks **B** Shape changes associated with the first PLS axes of two blocks: each diagram shows the block change along the PLS1 in the positive or negative direction, corresponding to Figure 3A.

PLS1 presented 87.22% of the total covariance, indicating that PLS1 represented the main covariance of two blocks. Figure 3A shows scatter plot of the PLS1 of two blocks; Figure 3B presents the shape changes of two blocks based on PLS1 scores. The shape variance of proximal part is more conservative with distal part variance. For PLS1, the pairwise correlation between blocks was up to 0.92 (P=0.0016, 10,000 permutation test rounds), as noted in the plots distributed around the diagonal line of the PLS1 scores coordinate in Figure 3A. However, the *RV* coefficient was only 0.44, indicating that the overall strength of association between blocks was relatively weak (P<0.001, 10,000 randomisation rounds). When the *RV* coefficient values are higher, the covariance of two blocks is stronger. Therefore, the null hypothesis was rejected. Although high correlation was noted between two blocks, the overall strength of association between blocks was weak. From the analysis results, the following was concluded: in beetle hind wings, the distal part, or more precisely the areas relevant to transverse folding (apical area, central area and radial cell of hind wing) exhibits both a certain degree of independence and a high correlation between other parts of wings in the total hind wing shape variances.

**Figure 4.**
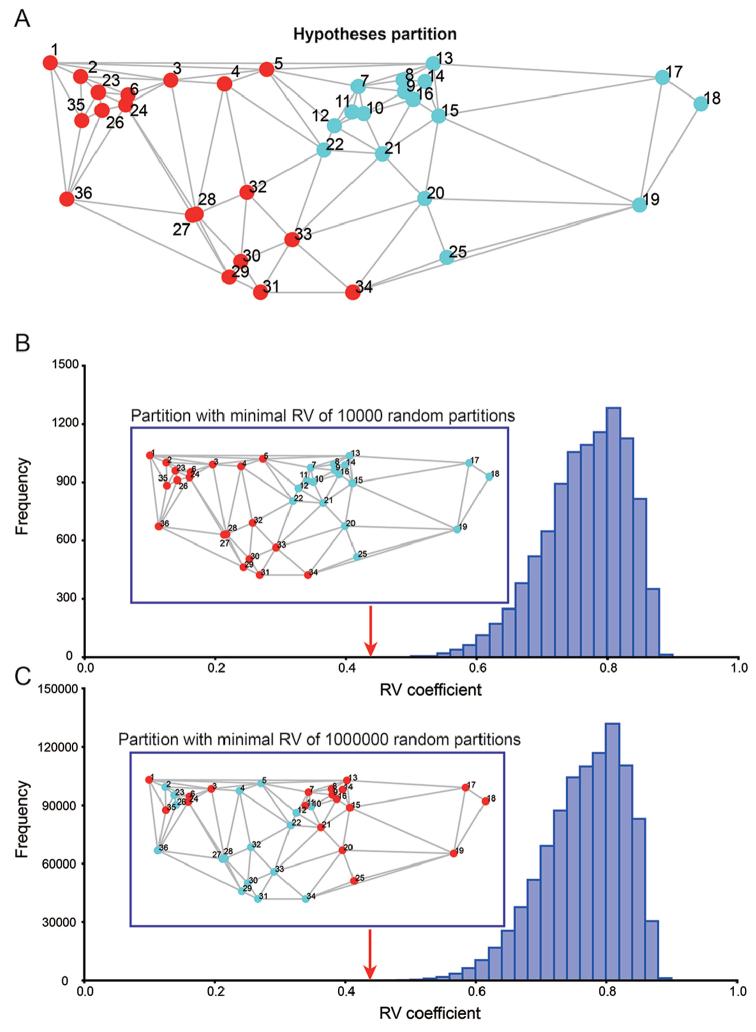
Modularity test results. **A** The hypothesized partition: proximal part landmarks 1-6, 23, 24, and 26–36 and distal part landmarks 7–22, 25; different colour presents different modules **B** The partition with minimal covariance in all evaluated 10^4^ partitions by *RV* coefficient **C** The partition with minimal covariance in all evaluated 10^6^ partitions by *RV* coefficient.

Based on the PLS analysis results, a modularity analysis was performed to evaluate whether the proximal and the distal parts of beetle hind wings are separate modules. In the same manner, the landmarks 7–22 and 25 involving transverse folding were extracted as the distal part and the remaining landmarks 1–6, 23–24 and 26–36 as the proximal part, which was our hypothesised partition (Figure 4A). Contiguous partitions were considered with 10,000 and 1,000,000 random partitions. The *RV* coefficient of the a priori hypothesis partition was 0.44, which was identical to that of the PLS analysis. The 10,000 random partition result showed that there are no partitions with an *RV* less than or equal to the a priori hypothesis. The minimal *RV* coefficient partition of 10,000 random partitions was our hypothesis (Figure 4B). Thus, our null hypothesis partition was not rejected; the proximal and distal parts of the hind wing had minimal covariance in all evaluated 10,000 partitions. The 1,000,000 random partition result revealed ten partitions with a *RV* less than or equal to the a priori hypothesis. The minimal *RV* coefficient of 1,000,000 random partitions was 0.41 (Figure 4C). The partition with minimal *RV* is presented in Figure 4C. However, our null hypothesis was still accepted, because the minimal *RV* was close to the *RV* of the hypothesis partition and because the partition with minimal *RV* was similar to our hypothesis partition. Therefore, the proximal and distal parts of the beetle’s hind wing are separate modules.

## Discussion

### Phylogenetic implications

The powerful visualisation tools of geometric morphometrics and the typical large amount of shape variables give rise to a specific exploratory style of analysis, allowing the identification and quantification of previously unknown shape features (Mitteroecker and Gunz 2009).

In this study, it was mainly focused on the wing variance of different species based on subtribe level of leaf beetles. Three main features of leaf beetle’s hind wing variance based on PCA results (Figure 2B) were concluded. All the three features were useful phylogenetic features on subtribe level by CVA testing. Based on PCV and CVA results, the apical area of hind wing presented large variance and could be useful morphological features to distinguishing the subtribe and Chrysomelina. The anal area was also useful to distinguishing the subtribe Doryphorina and . The changes of cross vein cv in the middle area were also useful in the subtribe level distinguishing.

The PCA results presented three phylogenetic features of hind wings of leaf beetles, and the changes of apical area was the most obvious (variance contribution ratio was 45.01%, Figure 2B). Subtribe beetles have a very short apical area in their hind wings (Figure 2C). Compare with , subtribe Chrysomelina beetles have a long and big apical area in their hind wings (Figure 2C). What is the role of apical area in wing evolution is still unclear. It’s worth to pay more attentions on the apical area of hind wings to explore answers.

### Functional and morphological implications

In our study, PLS analysis showed that the distal part of hind wing, which is involved in transverse folding and includes the apical area, central area, and radial cell, exhibited independent shape variance in the total variance of the hind wing (Figure 3). A modularity test was used to confirm that are the distal and proximal parts of the hind wing consisted of two modules (Figure 4). Modules are units exhibiting a high degree of integration from many or strong interactions but relative independence from other such units (Klingenberg 2008, 2009, 2011). For a morphometric analysis, these interactions should be manifested as strong covariation among parts within modules and weak covariation between modules (Klingenberg 2009, 2014); thus, the PLS analysis is reasonable in our study. The PLS analysis suggests that proximal and distal parts of chrysomelid hind wings have weak between-module integration (i.e., a low *RV* coefficient of 0.44) but strong within-module integration (i.e., a high correlation of 0.92). Based on the PLS analysis results, a modularity test to verify the hypothesis regarding partitioning as noted in Figure 4A was performed. The partition with a minimal *RV* coefficient of all evaluated partitions does fit with our hypothesised partition: the proximal and distal parts of hind wing are separate modules.

Various studies of *Drosophila*’s wings using morphometric approaches have addressed the question of whether anterior and posterior wing compartments are distinct modules reflecting phenotypic and genetic variation (Thompson and Woodruff 1982, Cavicchi et al. 1985, Cowley and Atchley 1990, Guerra et al. 1997, Baylac and Penin 1998, Klingenberg and Zaklan 2000). Based on correspondences to distinct cell lineages and domains of gene expression (Escoufier 1973, Lawrence and Morata 1976), the subdivision of wings into anterior and posterior compartments has been given special attention in *Drosophila*. However, Klingenberg and Zaklan (2000) used geometric morphometrics analysis and found that the covariation between landmarks in the anterior and posterior compartments are not weaker than expected for an arbitrary partition of the wing into two subsets of landmarks. This finding clearly contrasts with the conclusions of earlier studies that support the hypothesis that the anterior and posterior compartments are separate developmental modules.

What is the nature of the modularity interactions of hind wings? Generally, it can be developmental, functional, or genetic (Klingenberg 2008, 2009, 2011). Here, all of our hypotheses are based on the transverse folding function of the beetle hind wing. It’s thus concluded that the proximal and distal parts of hind wings are separate functional modules and that this separation is caused by the transverse folding function of a beetle’s hind wing. The veins of the distal part of the hind wings are mainly radial veins (RA_4_, RP, RP_2_ and radial cell, see Figure 1), and some veins of the proximal part are also radial veins (RA and radial bar, see Figure 1). Thus, the landmarks of both proximal and distal compartments are mainly derived from radial veins, indicating that the two compartments of hind wings are rarely separate developmental or genetic modules. This finding is likely related with the necessity to fold the hind wings transversely for complete storage below the elytra.

The separate modules of proximal and distal parts of Chrysomelid hind wings have been tested. The main reason of the separate modules could be attribute to its special function: transverse folding in hind wings of beetles. Although there were only 96 chrysomelid beetles considered in our study, it could be believed that other beetles feature the same modularity of hind wings, given that the transverse folding of the hind wing is a common feature of beetles. However, more data are needed to confirm this hypothesis. Here, the apical part of the hind wings of leaf beetles has an important influence on hind wing shape variance by PCA (Figure 2B). The shape variance of the apical part of the hind wing in all beetles (not exclusively leaf beetles) should be given more attention in studies of wings or flight. In particular, what effect could the relative size of the apical part have on beetle flying, folding function or phylogeny? What type of role does it play in wing evolution? These questions require more work to find the answers.

## Conclusion

Taking the PCA, CVA, PLS analyses and the modularity test into account, it was concluded that areas of the beetle hind wing relevant to transverse folding importantly influence hind wing shape variances and are relatively independent. In addition, the proximal and distal parts of a beetle’s hind wing are separate modules. The changes of apical area, anal area and middle area of hind wings were useful features to distinguishing subtribe level of leaf beetles. For beetles, hind wing folding is a morphological and functional compromise between fore wing evolution to elytra and the maintenance of hind wing flying function. This separate function modules could allow the hind wings to be folded at rest and to unfold when flying. In addition, the separate function could explain why beetles are the most prosperous animals in evolution. Our discovery could provide the theoretical basis and a new perspective for studies of the morphological evolution of wings and wing folding mechanisms.

## Author contributions

Experiments were planned by Jing Ren, Si-Qin Ge, and Run-Zhi Zhang. Species identification was completed by Si-Qin Ge. Experiments were conducted by Jing Ren. Analysis and interpretation of the results was performed by Jing Ren, Ming Bai, Run-Zhi Zhang, and Xing-Ke Yang. The paper was written by Jing Ren, Si-Qin Ge, and Run-Zhi Zhang.

## Conflict of interest

All authors declare there are no potential competing interests.

## Supplementary materials

Supplementary material 1 Images of hind wings. Data type: Species data This dataset is made available under the Open Database License (http://opendatacommons.org/licenses/odbl/1.0/). The Open Database License (ODbL) is a license agreement intended to allow users to freely share, modify, and use this Dataset while maintaining this same freedom for others, provided that the original source and author(s) are credited.Jing Ren, Ming Bai, Xing-Ke Yang, Run-Zhi Zhang, Si-Qin Ge

Supplementary material 2 Sample information. Data type: Specimens data This dataset is made available under the Open Database License (http://opendatacommons.org/licenses/odbl/1.0/). The Open Database License (ODbL) is a license agreement intended to allow users to freely share, modify, and use this Dataset while maintaining this same freedom for others, provided that the original source and author(s) are credited.Jing Ren, Ming Bai, Xing-Ke Yang, Run-Zhi Zhang, Si-Qin Ge

Supplementary material 3 Coordinates data of landmarks. Data type: Distribution data This dataset is made available under the Open Database License (http://opendatacommons.org/licenses/odbl/1.0/). The Open Database License (ODbL) is a license agreement intended to allow users to freely share, modify, and use this Dataset while maintaining this same freedom for others, provided that the original source and author(s) are credited.Jing Ren, Ming Bai, Xing-Ke Yang, Run-Zhi Zhang, Si-Qin Ge
