# Supplementary figures and images for: Geometric morphometrics analysis of the hind wing of leaf beetles: proximal and distal parts are separate modules
Source: Zookeys. 2017 Jul 20;(685):131–49. doi: 10.3897/zookeys.685.13084 (PMC5646652; doi:10.3897/zookeys.685.13084)

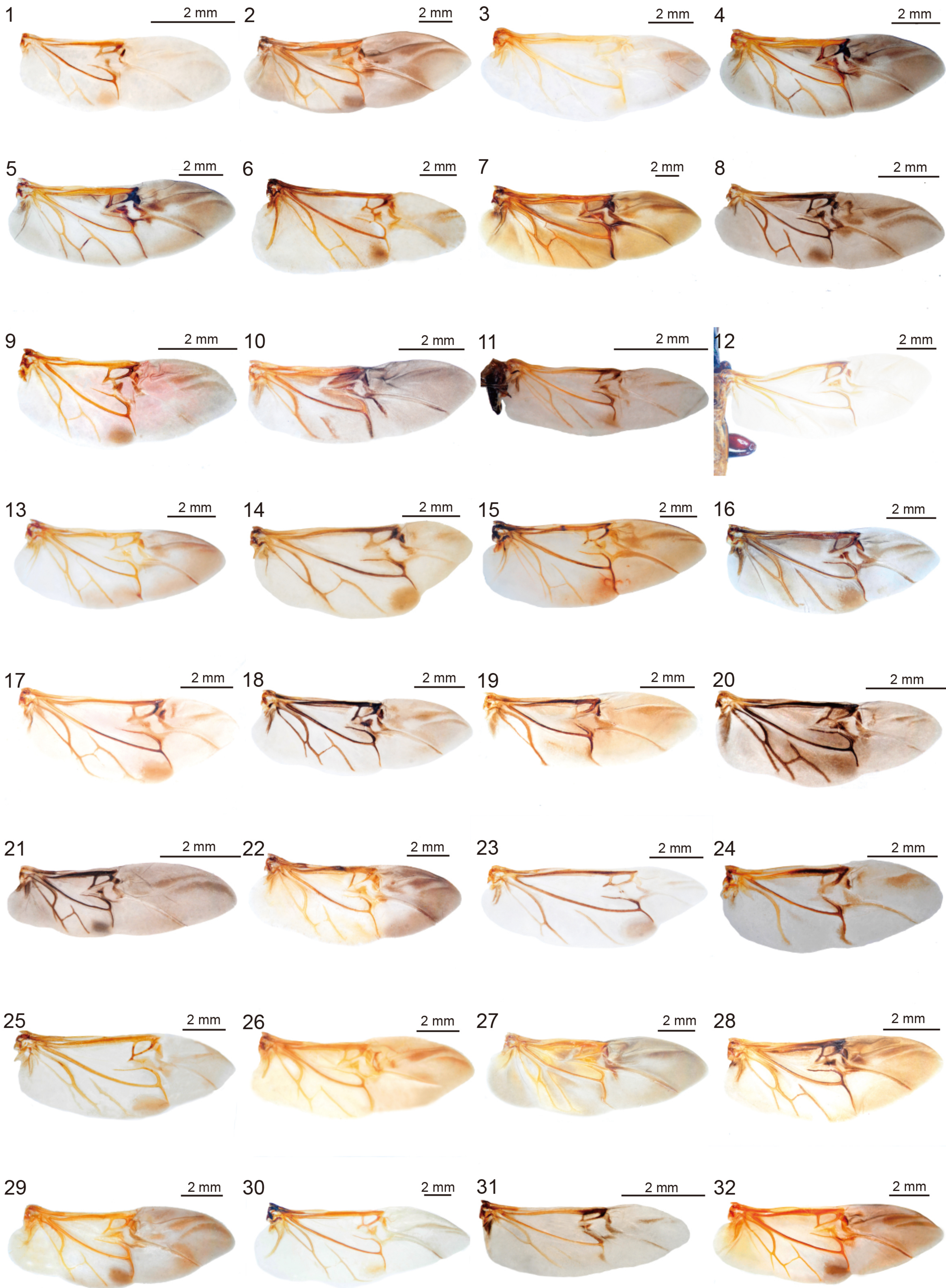

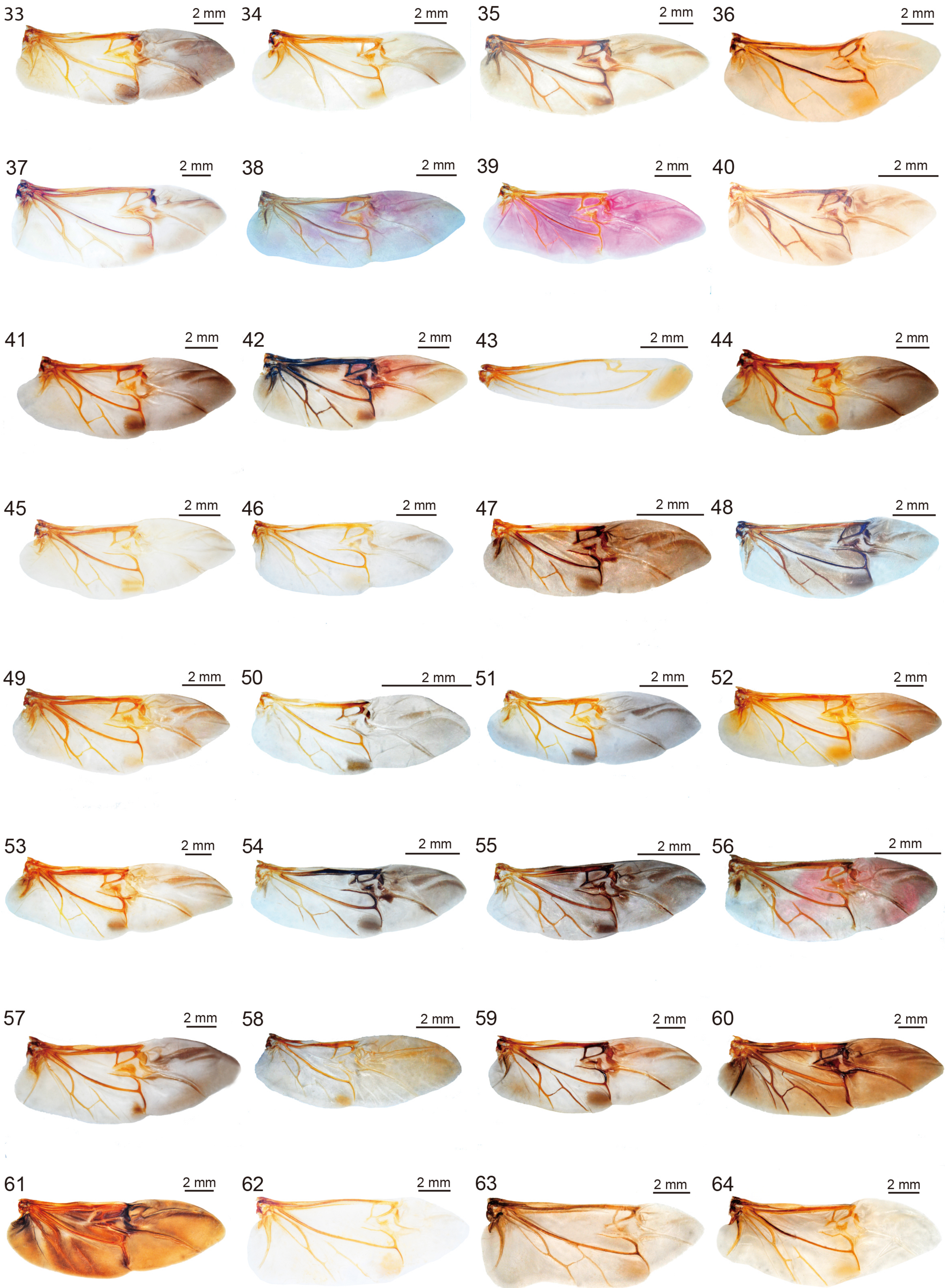

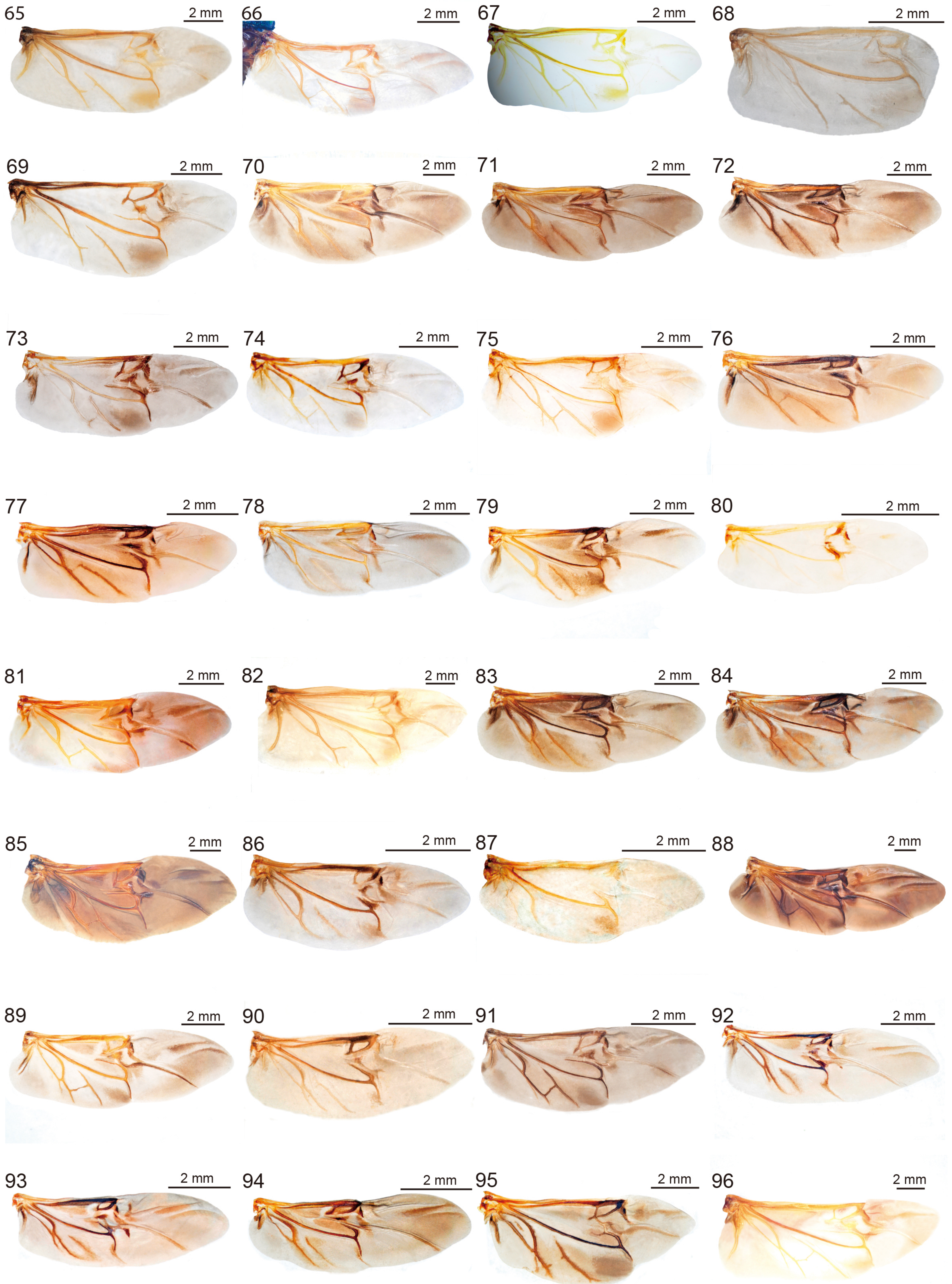

Supplement: Supplementary material 1 — Images of hind wings. [file zookeys-685-131-s001.pdf]
