# Supplementary material for: Geometric morphometrics analysis of the hind wing of leaf beetles: proximal and distal parts are separate modules
Source: Zookeys. 2017 Jul 20;(685):131–49. doi: 10.3897/zookeys.685.13084 (PMC5646652; doi:10.3897/zookeys.685.13084)
Supplement: Supplementary material 2 — Sample information. [file zookeys-685-131-s002.docx]

Supplementary file 2. Sample collection information

| **Sample #** | **Species** | **Subtribe** | **Collecting locations and time** |
| --- | --- | --- | --- |
| 1 | *Araucanomela wellingtonensis* Bechyné, 1973 | PHYLLODECTINA | Chile: Osorno: Antillanca |
| 2 | *Augomela hypochalcea* (Germar, 1848) | PHYLLODECTINA | Australia |
| 3 | *Calligrapha scalaris* (LeConte, 1824) | DORYPHORINA | America: Tian am bor |
| 4 | *Centroscelis laevigata* Achard, 1924 | GONIOCTENINA | New Zealand: Cape: Queenstown |
| 5 | *Centroscelis notala* Bechyné, 1946 | GONIOCTENINA | New Zealand: Cape: Somerset East, 1930.9 |
| 6 | *Oreina (Chrysochloa) cacaliae senecionis* (Schummel, 1843) | CHRYSOLININA | Poland |
| 7 | *Dicranosterna picea* Olivier, 1807 | DICRANOSTERNINA | Australia: Moreton Bay |
| 8 | *Novacastria nothofagi* (Reid, 2002) | - | Australia: Cradle Mountain, 1998.11.30 |
| 9 | *Gavirga monrosi* Bechyné, 1950 | PHYLLODECTINA | Paraguay |
| 10 | *Hispostoma marginatum* Weise, 1907 | HISPOSTOMINA | Belgian Congo: Elizabethville, 1927 |
| 11 | *Hydrothassa marginella* (Linnaeus,1758) | CHRYSOMELINA | Slovakia Sahy, 1937 |
| 12 | *Johannica gemellata* (Westwood, 1849) | PHYLLOCHARINA | Australia: Queensland |
| 13 | *Labidomera clivicollis* (Kirby, 1937) | DORYPHORINA | America: Bethel Me |
| 14 | *Lioplacis elliptica*(Stål, 1860) | DICRANOSTERNINA | Argentina: Santiago del Estero |
| 15 | [*Leptinotarsa decemlineata*](http://apps.webofknowledge.com/full_record.do?product=UA&search_mode=GeneralSearch&qid=67&SID=S1NcQiqikSQn6s1vNjK&page=1&doc=6) (Say, 1824) | DORYPHORINA | Albania: Kor |
| 16 | *Ceralces apicatus* Fairmaire, 1884 | - | Ethiopia: Sidamo, 1997.5.7 |
| 17 | *Lioplacis sp.* | DICRANOSTERNINA | Brazil: Nova Teutonia, 1934.10.8 |
| 18 | *Gonioctena* *lineata* (Genè, 1839) | GONIOCTENINA | Sardegna (NU) Fonni: Punra Ninnieri |
| 19 | *Mesoplatys ochroptera* (Stål, 1857) | CHRYSOMELINA | Congo belge: P.N.U.Mabwe, 1949.2.2 |
| 20 | *Microtheca boliviana* Achard, 1917 | ENTOMOSCELINA | Uruguay: Lavalleja: Artigas Pampa, 2001.9.28 |
| 21 | *Novacastria nothofagi* (Reid, 2002) | - | Australia: New South Wales, 1999.12.29 |
| 22 | *Oidosoma ornatum* (Baly, 1860) | PHYLLOCHARINA | North-Western Rhodesia: Iusemzka, 1916.7 |
| 23 | *Peltoschema nigroconspersa* Clark, 1865 | - | Western Australia: Kenwick, 1960.9 |
| 24 | *Phaedonia circumcincta* Berti, 1968 | CHRYSOMELINA | Burundi: Bujumbura R.P.Giraudin, 1969.9.3 |
| 25 | *Phyllocharis*  *undulata* (Linnaeus, 1758) | PHYLLOCHARINA | Vietnam: Tonkin |
| 26 | *Pixis clavigera* Stål, 1860 | PHYLLODECTINA | Brazil: Rio de Janeiro: Corccovado Guanabara, 1957.11 |
| 27 | *Plagiodera tarsata* Duvivier, 1891 | CHRYSOMELINA | Madagascar: Tamatave: Moramange, 1996.12.22 |
| 28 | *Planagetes proteus* (Stål, 1860) | PHYLLODECTINA | Brazil: Gerais: Pirapora, 1905.11 |
| 29 | *Platymela sticticollis* Baly, 1856 | PHYLLODECTINA | Australia: Cape York |
| 30 | *Platyphora pura* (Stål, 1858) | DORYPHORINA | South Africa: Zululand: Santa Lucia Bay |
| 31 | *Prasocuris phellandrii* (Linnaeus, 1758) | CHRYSOMELINA | France |
| 32 | *Procrisina pictipennis* (Boheman, 1859) | PAROPSINA | Australia: New South Wales, 1958.10 |
| 33 | *Augomela splendens* (Macleay, 1826) | PHYLLODECTINA | New Guinea |
| 34 | *Sphaeratrix latifrons gabonensis* Bechyné, 1948 | MONARDITINA | Tanzania, 1995.1.10 |
| 35 | *Sterromela nucea* (Erichson, 1842) | DICRANOSTERNINA | England: North Lincolnshire: New Holland |
| 36 | *Zygogramma suturalis* (Fabricius, 1775) | DORYPHORINA | America: New Jersey |
| 37 | *Aesernoides nigrofasciata* Jacoby, 1885 | PHYLLODECTINA | Australia: New South Wales: Lismore, 1903.11.28 |
| 38 | *Calomela bartoni* Froggatt, 1989 | PHYLLODECTINA | Australia: New South Wales: Captains Flat, 1978.4.20 |
| 39 | *Carystea imperialis* (Blackburn, 1893) | CHRYSOMELINA | Australia: Finke Gorge National Park, 1974.1.28 |
| 40 | *Chalcolampra* sp. | PHYLLOCHARINA | Australia: Queensland: Eimeo, 1976 |
| 41 | *Chalcomela* sp. | CHRYSOMELINA | Canada: Oshawa: Russett Park, 1988.2.20 |
| 42 | *Chrysophtharta agricola* (Chapuis, 1877) | PAROPSINA | Australia: Tasmania: Liffey Valley, 1981.11.12 |
| 43 | *Clidonotus gibbosa* (Baly 1862) | PHYLLODECTINA | Australia: New South Wales: Richmond, 1909 |
| 44 | *Cyclomela nitida* (Baly 1856) | CHRYSOMELINA | England: Surrey: Elstead, 1978.10.21 |
| 45 | *Diacosma tricolor* Lhoste, 1934 | CHRYSOMELINA | Australia: Madura, 1968.4.30 |
| 46 | *Eulina curtisi* Baly, 1855 | PHYLLOCHARINA | Australia: Queensland |
| 47 | *Faex orphana* (Erichson, 1842) | PAROPSINA | Australia: Victoria: Morwell |
| 48 | *Lamprolina impressicollis* Baly, 1856 | ENTOMOSCELINA | Australia: New South Wales |
| 49 | *Macelola geniculata* (Baly, 1856) | PHYLLODECTINA | Australia: Northern Territory: Tennant Creek, 1979.11.10 |
| 50 | *Oomela* sp. | PHYLLOCHARINA | Australia: Queensland: Heathlands Regional Park, 1992.1.20 |
| 51 | *Paropsimorpha caudate* (Blackburn, 1892) | CHRYSOMELINA | Australia: Queensland: Paluma, 1969.1.9 |
| 52 | *Paropsis atomaria* Olivier, 1807 | PAROPSINA | Australia: Canberra, 1967.1 |
| 53 | *Paropsisterna variicollis* (Chapuis, 1877) | PAROPSINA | America: New Hampshire: Waterville Valley, |
| 54 | *Peltoschema turbata* Chapius, 1877 | - | Australia: Geraldton, 1926.9 |
| 55 | *Philhydronopa* sp*.* | PAROPSINA | South Australia: Morgan, 1970.12.16 |
| 56 | *Starycea jansoni* (Baly, 1865) | CHRYSOMELINA | Australia: Millstream Chichester National Park, 1970.10.25 |
| 57 | *Stethomela* sp. | PHYLLODECTINA | Australia: Queensland |
| 58 | *Tinosis* sp*.* | PHYLLODECTINA | Australia: Queensland: Mount Bartle Frere, 2009.11.19 |
| 59 | *Trachymela regularis* (Blackburn, 1892) | PAROPSINA | Australia: New South Wales: Dainers Gap, 1973.3.9 |
| 60 | *Trochalodes circe* (Stal, 1860) | DICRANOSTERNINA | Australia: Queensland: Mount Walsh National Park, 1972.4.1 |
| 61 | *Agasta formosa* Hope, 1840 | CHRYSOMELINA | China: Yunnan: Xishuangbanna, 1959.5.5 |
| 62 | *Agrosteella punctata* Ge *et* Yang, 2002 | - | China: Tibet: Moto, 1980.5.22 |
| 63 | *Agrosteomela indica* (Hope, 1931) | CHRYSOLININA | China: Sichuan: Emei Mountain, 1957.6.28 |
| 64 | *Ambrostoma fortune* (Baly, 1860) | CHRYSOLININA | China: Fujian: Jiuqu, 1982.6. |
| 65 | *Ambrostoma superbum* (Thunberg, 1787) | CHRYSOLININA | China: Heilongjiang: Wudalianchi, 1971.7.14 |
| 66 | *Paropsides pardalis*Jacoby, 1892 | PAROPSINA | China: Yunnan: Pingbiandaweishan, 1956.6.17 |
| 67 | *Chrysolina aeruginosa* (Faldermann, 1835) | CHRYSOLININA | China: Xinjiang, 1992.8.11 |
| 68 | *Chrysolina aeruginosa* (Faldermann, 1835) | CHRYSOLININA | China: Sichuan: Litang, 1982.6.4 |
| 69 | *Chrysolina aurichalcea* (Mannerheim, 1825) | CHRYSOLININA | China: Fujian: Jianyang, 1960.4.30 |
| 70 | *Chrysomela populi* Linnaeus, 1758 | CHRYSOMELINA | China: Hubei: Xingshanlongmen River, 1993.6.22 |
| 71 | *Chrysomela salicivorax* (Fairmaire, 1888) | CHRYSOMELINA | China: Beijing: Badaling, 1964.6.4 |
| 72 | *Chrysomela vigintipunctata* (Scopoli, 1763) | CHRYSOMELINA | China: Yunnan: Xiaozhongdian, 1984.7.31 |
| 73 | *Colaphellus*  *bowringii* (Baly, 1860) | CHRYSOMELINA | China: Shanghai, 1954.11.4 |
| 74 | *Entomoscelis orientalis* Motschulsky, 1860 | ENTOMOSCELINA | China: Beijing, 1962.5.31 |
| 75 | *Entomoscelis pulla* Daccordi *et* Ge, 2009 | ENTOMOSCELINA | China: Beijing, 1950.5.6 |
| 76 | *Gastrolina depressa* Baly, 1859 | CHRYSOMELINA | China: Sichuan: Pengshui, 1988.7.14 |
| 77 | *Gastrolina tonkinea* Chen, 1931 | CHRYSOMELINA | China: Yunnan: Xishuangbanna, 1959.6.26 |
| 78 | *Gastrolinoides japonica* Kimoto, 1962 | CHRYSOMELINA | China: Hubei: Xingshanlongmen River, 1993.6.23 |
| 79 | *Gastrophysa atrocyanea* Motschulsky, 1860 | CHRYSOMELINA | China: Sichuan: Fengjie, 1994.5.23 |
| 80 | *Gastrophysa mannerheimi* (Stål, 1858) | CHRYSOMELINA | China: Inner Mongolia, 1971.6.29 |
| 81 | *Gonioctena tredecimmaculata* (Jacoby, 1888) | GONIOCTENINA | China: Hunan: Tianping Mountain, 1988.8.12 |
| 82 | *Humba cyanicollis*  (Hope, 1831) | CHRYSOLININA | China: Sichuan: Emei Mountain, 1992.8.2 |
| 83 | *Linaeidea aenea* (Linaeidea, 1758 ) | CHRYSOMELINA | China: Jilin: Changbai Mountain, 1987.7.18 |
| 84 | *Linaeidea placida* (Chen, 1934) | CHRYSOMELINA | China: Sichuan: Baoguosi, 1957.4.20 |
|  |  |  |  |
| 85 | *Lycaria westermanni* Stål, 1857 | PHYLLOCHARINA | Vietnam: Tonkin |
| 86 | *Phaedon pyritosus* (Rossi, 1792) | CHRYSOMELINA | Turkmenistan, 1994.5 |
| 87 | *Odontoedon fulvescens* (Weise,1922) | - | China: Guangxi: Guilinyan Mountain, 1983.6.24 |
| 88 | *Paropsides nigrosparsus* Fairmaire, 1889 | PAROPSINA | China: Guangxi: Longtengbaiyan Mountain, 1963.6.21 |
| 89 | *Paropsides soriculata* (Swartz, 1808) | PAROPSINA | China: Yunnan: Menglongbanna, 1958.4.27 |
| 90 | *Phaedon brassicae* Baly, 1874 | CHRYSOMELINA | China: Fujian: Dehua, 1960.6.5 |
| 91 | *Phola octodecimguttata* (Fabricius, 1775) | PHYLLOCHARINA | China: Guangxi: Ling Mountain, 1984.6.3 |
| 92 | *Phratora bicolor* Gressitt *et* Kimoto, 1963 | PHYLLODECTINA | China: Sichuan: Taocheng Haizi Mountain, 1982.6.10 |
| 93 | *Phratora laticollis* (Suffrian 1851) | PHYLLODECTINA | China: Sichuan: Kangding, 1983.6.24 |
| 94 | *Plagiodera versicolor*(Laicharting, 1781) | CHRYSOMELINA | China: Yunnan: Lijiang, 1981.7.30 |
| 95 | *Potaninia assamensis* (Baly, 1879) | ENTOMOSCELINA | China: Guizhou: Xishui, 2000.9.28 |
| 96 | *Spherolina sp.* | CHRYSOLININA | India: Kodaikanal: Pulney Hills, 1953.5 |

Table S1. Subtribe information followed that of Seeno and Wilcox (1982).
