# Supplementary material for: Geometric morphometrics analysis of the hind wing of leaf beetles: proximal and distal parts are separate modules
Source: Zookeys. 2017 Jul 20;(685):131–49. doi: 10.3897/zookeys.685.13084 (PMC5646652; doi:10.3897/zookeys.685.13084)
Supplement: Supplementary material 3 — Coordinates data of landmarks. [file zookeys-685-131-s003.docx]

Supplementary file 3. Landmark coordinate data of 96 specimens

LM=36

324.00000 2027.00000

457.00000 1970.00000

782.00000 1922.00000

1242.00000 1877.00000

1324.00000 1925.00000

681.00000 1865.00000

1786.00000 1781.00000

2020.00000 1803.00000

2003.00000 1747.00000

1780.00000 1689.00000

1754.00000 1708.00000

1639.00000 1606.00000

2148.00000 1867.00000

2131.00000 1792.00000

2133.00000 1627.00000

2011.00000 1704.00000

3544.00000 1732.00000

3923.00000 1421.00000

3397.00000 800.00000

1981.00000 1175.00000

1821.00000 1391.00000

1495.00000 1475.00000

542.00000 1914.00000

681.00000 1820.00000

2058.00000 843.00000

552.00000 1829.00000

951.00000 1216.00000

951.00000 1216.00000

1178.00000 850.00000

1144.00000 910.00000

1180.00000 850.00000

1215.00000 1276.00000

1328.00000 1051.00000

1596.00000 755.00000

422.00000 1766.00000

392.00000 1611.00000

IMAGE=Araucanomela wellingtonensis.tif

ID=1

SCALE=0.001313

LM=36

397.00000 2322.00000

563.00000 2200.00000

1091.00000 2047.00000

1306.00000 2016.00000

1436.00000 2060.00000

822.00000 2034.00000

2029.00000 1915.00000

2260.00000 1948.00000

2270.00000 1884.00000

2066.00000 1746.00000

2003.00000 1759.00000

1884.00000 1653.00000

2410.00000 2039.00000

2382.00000 1933.00000

2462.00000 1772.00000

2319.00000 1866.00000

3701.00000 1872.00000

4066.00000 1665.00000

3559.00000 1168.00000

2339.00000 1240.00000

2121.00000 1534.00000

1749.00000 1558.00000

638.00000 2075.00000

801.00000 1958.00000

2384.00000 947.00000

638.00000 1975.00000

1056.00000 1271.00000

1056.00000 1271.00000

1266.00000 944.00000

1194.00000 1075.00000

1270.00000 940.00000

1366.00000 1413.00000

1483.00000 1230.00000

1842.00000 837.00000

501.00000 1954.00000

297.00000 1554.00000

IMAGE=Augomela hypochalcae.tif

ID=2

SCALE=0.002642

LM=36

352.00000 2151.00000

518.00000 2082.00000

1049.00000 2023.00000

1363.00000 1975.00000

1649.00000 2030.00000

776.00000 1944.00000

2208.00000 1872.00000

2511.00000 1885.00000

2515.00000 1816.00000

2246.00000 1727.00000

2180.00000 1727.00000

2132.00000 1685.00000

2725.00000 1944.00000

2628.00000 1858.00000

2687.00000 1709.00000

2594.00000 1754.00000

3866.00000 1720.00000

4153.00000 1534.00000

3818.00000 996.00000

2594.00000 1120.00000

2380.00000 1427.00000

1935.00000 1471.00000

607.00000 1975.00000

756.00000 1885.00000

2670.00000 809.00000

625.00000 1865.00000

1132.00000 1127.00000

1132.00000 1127.00000

1487.00000 651.00000

1332.00000 827.00000

1490.00000 654.00000

1452.00000 1299.00000

1718.00000 985.00000

2104.00000 585.00000

518.00000 1837.00000

380.00000 1496.00000

IMAGE=Calligrapha scalavis.tif

ID=3

SCALE=0.002444

LM=36

366.00000 2082.00000

566.00000 1985.00000

1121.00000 1930.00000

1394.00000 1885.00000

1659.00000 1937.00000

845.00000 1837.00000

2018.00000 1775.00000

2263.00000 1809.00000

2266.00000 1744.00000

2035.00000 1606.00000

1980.00000 1620.00000

1873.00000 1540.00000

2484.00000 1909.00000

2415.00000 1789.00000

2470.00000 1592.00000

2339.00000 1689.00000

3707.00000 1785.00000

4049.00000 1547.00000

3594.00000 940.00000

2339.00000 1123.00000

2142.00000 1385.00000

1770.00000 1465.00000

666.00000 1878.00000

825.00000 1782.00000

2521.00000 647.00000

676.00000 1768.00000

1056.00000 1102.00000

1121.00000 1096.00000

1114.00000 716.00000

1339.00000 851.00000

1428.00000 654.00000

1494.00000 1227.00000

1611.00000 1065.00000

2018.00000 568.00000

566.00000 1751.00000

366.00000 1292.00000

IMAGE=Centroscelis laevigata.tif

ID=4

SCALE=0.002363

LM=36

307.00000 2085.00000

497.00000 1961.00000

1083.00000 1920.00000

1342.00000 1878.00000

1525.00000 1937.00000

780.00000 1834.00000

2008.00000 1796.00000

2242.00000 1803.00000

2242.00000 1751.00000

2004.00000 1637.00000

1952.00000 1640.00000

1839.00000 1568.00000

2449.00000 1916.00000

2401.00000 1806.00000

2466.00000 1620.00000

2321.00000 1696.00000

3728.00000 1878.00000

4104.00000 1575.00000

3594.00000 1034.00000

2346.00000 1151.00000

2128.00000 1399.00000

1756.00000 1471.00000

597.00000 1868.00000

766.00000 1778.00000

2587.00000 682.00000

597.00000 1751.00000

1025.00000 1102.00000

1108.00000 1116.00000

1163.00000 671.00000

1380.00000 813.00000

1466.00000 623.00000

1418.00000 1271.00000

1621.00000 1054.00000

2121.00000 558.00000

476.00000 1689.00000

370.00000 1254.00000

IMAGE=Centroscelis ornata.tif

ID=5

SCALE=0.002558

LM=36

539.00000 2252.00000

713.00000 2183.00000

1200.00000 2078.00000

1603.00000 2003.00000

1742.00000 2067.00000

944.00000 2044.00000

2355.00000 1907.00000

2597.00000 1907.00000

2600.00000 1847.00000

2403.00000 1725.00000

2292.00000 1779.00000

2209.00000 1719.00000

2748.00000 1961.00000

2688.00000 1884.00000

2802.00000 1670.00000

2634.00000 1784.00000

3903.00000 1697.00000

4002.00000 1602.00000

3737.00000 1069.00000

2631.00000 1214.00000

2440.00000 1514.00000

2095.00000 1559.00000

799.00000 2101.00000

924.00000 2004.00000

2697.00000 912.00000

821.00000 1981.00000

1366.00000 1288.00000

1366.00000 1288.00000

1826.00000 654.00000

1634.00000 969.00000

1825.00000 653.00000

1562.00000 1459.00000

1890.00000 1100.00000

2241.00000 693.00000

710.00000 1970.00000

476.00000 1006.00000

IMAGE=Chrysochloa cacaliae senecionis.tif

ID=6

SCALE=0.003078

LM=36

314.00000 2089.00000

511.00000 1981.00000

1132.00000 1904.00000

1314.00000 1844.00000

1605.00000 1918.00000

821.00000 1810.00000

2107.00000 1756.00000

2409.00000 1796.00000

2403.00000 1722.00000

2181.00000 1605.00000

2092.00000 1613.00000

1941.00000 1508.00000

2651.00000 1924.00000

2551.00000 1810.00000

2617.00000 1576.00000

2472.00000 1679.00000

4019.00000 1656.00000

4219.00000 1511.00000

3737.00000 932.00000

2540.00000 1092.00000

2329.00000 1380.00000

1873.00000 1453.00000

605.00000 1853.00000

784.00000 1745.00000

2665.00000 661.00000

617.00000 1747.00000

930.00000 1251.00000

1106.00000 1123.00000

1169.00000 642.00000

1326.00000 904.00000

1471.00000 587.00000

1297.00000 1303.00000

1514.00000 1115.00000

2187.00000 530.00000

536.00000 1673.00000

328.00000 1189.00000

IMAGE=Dicranosterna picea.tif

ID=7

SCALE=0.004525

LM=36

368.00000 2243.00000

496.00000 2155.00000

972.00000 2075.00000

1309.00000 2041.00000

1394.00000 2084.00000

744.00000 2024.00000

1842.00000 1961.00000

2078.00000 1970.00000

2087.00000 1913.00000

1876.00000 1804.00000

1796.00000 1819.00000

1713.00000 1710.00000

2269.00000 2061.00000

2224.00000 1958.00000

2244.00000 1787.00000

2135.00000 1876.00000

3643.00000 1833.00000

4122.00000 1400.00000

3435.00000 929.00000

2112.00000 1280.00000

1950.00000 1539.00000

1528.00000 1631.00000

571.00000 2064.00000

719.00000 1975.00000

2178.00000 892.00000

573.00000 1978.00000

955.00000 1408.00000

955.00000 1408.00000

1175.00000 944.00000

1155.00000 1026.00000

1178.00000 952.00000

1306.00000 1417.00000

1437.00000 1146.00000

1614.00000 838.00000

505.00000 1961.00000

417.00000 1688.00000

IMAGE=Ewanius nothofagi.tif

ID=8

SCALE=0.001819

LM=36

354.00000 2149.00000

516.00000 2061.00000

987.00000 1987.00000

1312.00000 1930.00000

1360.00000 1970.00000

776.00000 1927.00000

1939.00000 1782.00000

2252.00000 1802.00000

2235.00000 1739.00000

1970.00000 1633.00000

1887.00000 1656.00000

1782.00000 1565.00000

2443.00000 1893.00000

2366.00000 1753.00000

2397.00000 1545.00000

2258.00000 1665.00000

3731.00000 1722.00000

4105.00000 1380.00000

3478.00000 770.00000

2241.00000 944.00000

1984.00000 1337.00000

1545.00000 1457.00000

619.00000 1964.00000

761.00000 1878.00000

2383.00000 593.00000

613.00000 1850.00000

987.00000 1123.00000

987.00000 1123.00000

1007.00000 781.00000

1109.00000 881.00000

1186.00000 585.00000

1277.00000 1283.00000

1426.00000 1001.00000

1728.00000 522.00000

502.00000 1850.00000

405.00000 1474.00000

IMAGE=Gavirga monrosi.tif

ID=9

SCALE=0.001397

LM=36

172.00000 2106.00000

351.00000 2007.00000

917.00000 1971.00000

1133.00000 1927.00000

1527.00000 1985.00000

641.00000 1896.00000

1824.00000 1825.00000

2024.00000 1861.00000

2024.00000 1861.00000

1866.00000 1750.00000

1800.00000 1769.00000

1583.00000 1700.00000

2238.00000 1952.00000

2219.00000 1896.00000

2283.00000 1658.00000

2120.00000 1783.00000

3807.00000 1995.00000

4017.00000 1865.00000

3694.00000 1182.00000

2212.00000 1208.00000

1894.00000 1498.00000

1897.00000 1510.00000

465.00000 1912.00000

629.00000 1828.00000

2347.00000 756.00000

455.00000 1797.00000

1002.00000 1154.00000

1002.00000 1154.00000

1362.00000 753.00000

1362.00000 753.00000

1362.00000 753.00000

1440.00000 1106.00000

1706.00000 724.00000

1706.00000 728.00000

384.00000 1766.00000

274.00000 1312.00000

IMAGE=Hispostoma marginatum.tif

ID=10

SCALE=0.001696

LM=36

563.00000 2292.00000

679.00000 2247.00000

1143.00000 2124.00000

1437.00000 2073.00000

1807.00000 2134.00000

919.00000 2096.00000

2271.00000 2030.00000

2460.00000 2030.00000

2429.00000 1959.00000

2222.00000 1875.00000

2182.00000 1903.00000

2106.00000 1842.00000

2657.00000 2058.00000

2554.00000 1985.00000

2587.00000 1837.00000

2474.00000 1915.00000

3941.00000 1835.00000

4035.00000 1707.00000

3675.00000 1215.00000

2422.00000 1462.00000

2283.00000 1660.00000

2047.00000 1707.00000

771.00000 2141.00000

896.00000 2061.00000

2455.00000 1156.00000

792.00000 2044.00000

1301.00000 1493.00000

1301.00000 1493.00000

1378.00000 1286.00000

1689.00000 1033.00000

1695.00000 1031.00000

1510.00000 1552.00000

2050.00000 1039.00000

2050.00000 1039.00000

684.00000 1974.00000

698.00000 1684.00000

IMAGE=Hydrothassa mavginella.tif

ID=11

SCALE=0.001159

LM=36

151.00000 2007.00000

330.00000 1985.00000

1002.00000 1882.00000

1206.00000 1879.00000

1666.00000 2089.00000

611.00000 1865.00000

2243.00000 2063.00000

2434.00000 2087.00000

2455.00000 2042.00000

2198.00000 1861.00000

2170.00000 1858.00000

2059.00000 1780.00000

2594.00000 2162.00000

2533.00000 2084.00000

2620.00000 1886.00000

2476.00000 1985.00000

3689.00000 2266.00000

3990.00000 2122.00000

3805.00000 1387.00000

2617.00000 1328.00000

2264.00000 1639.00000

1930.00000 1592.00000

436.00000 1863.00000

596.00000 1802.00000

2738.00000 963.00000

460.00000 1785.00000

1230.00000 1232.00000

1230.00000 1232.00000

1475.00000 867.00000

1541.00000 1050.00000

1659.00000 789.00000

1473.00000 1373.00000

1713.00000 1199.00000

2196.00000 798.00000

373.00000 1729.00000

486.00000 1324.00000

IMAGE=Johannica gemellata.tif

ID=12

SCALE=0.002628

LM=36

490.00000 2220.00000

671.00000 2076.00000

1176.00000 2020.00000

1402.00000 1960.00000

1740.00000 2007.00000

916.00000 1957.00000

2107.00000 1869.00000

2374.00000 1904.00000

2399.00000 1832.00000

2164.00000 1716.00000

2079.00000 1738.00000

2001.00000 1662.00000

2543.00000 1979.00000

2518.00000 1869.00000

2621.00000 1669.00000

2440.00000 1775.00000

3860.00000 1738.00000

3966.00000 1631.00000

3612.00000 1017.00000

2462.00000 1164.00000

2239.00000 1459.00000

1856.00000 1534.00000

734.00000 2004.00000

878.00000 1891.00000

2521.00000 791.00000

750.00000 1885.00000

1120.00000 1245.00000

1139.00000 1245.00000

1226.00000 816.00000

1355.00000 901.00000

1458.00000 697.00000

1389.00000 1412.00000

1618.00000 1104.00000

2016.00000 650.00000

628.00000 1782.00000

443.00000 1245.00000

IMAGE=Labidomera clivicollis.tif

ID=13

SCALE=0.002231

LM=36

306.00000 2040.00000

461.00000 1976.00000

1070.00000 1960.00000

1414.00000 1963.00000

1722.00000 2032.00000

773.00000 1859.00000

2446.00000 1930.00000

2762.00000 1981.00000

2757.00000 1898.00000

2526.00000 1767.00000

2409.00000 1787.00000

2272.00000 1665.00000

2902.00000 2052.00000

2893.00000 1967.00000

2993.00000 1782.00000

2808.00000 1859.00000

3954.00000 1961.00000

4076.00000 1847.00000

3908.00000 1397.00000

2985.00000 1212.00000

2634.00000 1491.00000

2192.00000 1459.00000

591.00000 1875.00000

761.00000 1821.00000

3184.00000 944.00000

628.00000 1747.00000

1309.00000 1046.00000

1309.00000 1046.00000

1417.00000 878.00000

1548.00000 850.00000

1725.00000 576.00000

1691.00000 1274.00000

1879.00000 1106.00000

2648.00000 539.00000

514.00000 1668.00000

542.00000 1203.00000

IMAGE=Leioplacis clliptica.tif

ID=14

SCALE=0.001990

LM=36

259.00000 2601.00000

509.00000 2488.00000

1108.00000 2457.00000

1427.00000 2419.00000

1741.00000 2541.00000

854.00000 2381.00000

2311.00000 2389.00000

2633.00000 2454.00000

2667.00000 2363.00000

2425.00000 2207.00000

2303.00000 2222.00000

2155.00000 2093.00000

2842.00000 2564.00000

2762.00000 2446.00000

2910.00000 2177.00000

2732.00000 2298.00000

4629.00000 2260.00000

4750.00000 2150.00000

4230.00000 1513.00000

2789.00000 1600.00000

2588.00000 1922.00000

2098.00000 1968.00000

634.00000 2385.00000

816.00000 2268.00000

2899.00000 1118.00000

687.00000 2241.00000

1237.00000 1471.00000

1275.00000 1479.00000

1378.00000 917.00000

1457.00000 1194.00000

1518.00000 891.00000

1514.00000 1691.00000

1859.00000 1289.00000

2182.00000 864.00000

486.00000 2165.00000

380.00000 1494.00000

IMAGE=Leptinotarsa decimlineata.tif

ID=15

SCALE=0.002541

LM=36

320.00000 2171.00000

498.00000 2070.00000

985.00000 2029.00000

1356.00000 1998.00000

1516.00000 2049.00000

783.00000 1941.00000

2286.00000 1907.00000

2506.00000 1923.00000

2503.00000 1855.00000

2304.00000 1718.00000

2218.00000 1728.00000

2115.00000 1666.00000

2667.00000 1998.00000

2607.00000 1902.00000

2734.00000 1692.00000

2558.00000 1803.00000

3856.00000 1780.00000

4001.00000 1643.00000

3646.00000 1127.00000

2594.00000 1220.00000

2418.00000 1485.00000

2011.00000 1490.00000

609.00000 1961.00000

765.00000 1891.00000

2791.00000 795.00000

635.00000 1853.00000

1234.00000 1184.00000

1242.00000 1181.00000

1542.00000 718.00000

1542.00000 718.00000

1542.00000 718.00000

1506.00000 1376.00000

2146.00000 643.00000

2146.00000 643.00000

498.00000 1796.00000

485.00000 1275.00000

IMAGE=Leralces apicatus.tif

ID=16

SCALE=0.002245

LM=36

311.00000 2244.00000

477.00000 2189.00000

1027.00000 2106.00000

1407.00000 2060.00000

1560.00000 2101.00000

762.00000 2060.00000

2322.00000 1928.00000

2667.00000 1959.00000

2651.00000 1866.00000

2415.00000 1749.00000

2330.00000 1780.00000

2172.00000 1689.00000

2851.00000 2044.00000

2781.00000 1928.00000

2835.00000 1692.00000

2672.00000 1809.00000

3783.00000 1829.00000

4063.00000 1687.00000

3825.00000 1194.00000

2781.00000 1171.00000

2485.00000 1487.00000

2011.00000 1555.00000

596.00000 2099.00000

754.00000 2018.00000

2975.00000 839.00000

615.00000 1961.00000

1143.00000 1280.00000

1143.00000 1280.00000

1192.00000 1127.00000

1348.00000 1031.00000

1545.00000 731.00000

1490.00000 1469.00000

1685.00000 1226.00000

2286.00000 681.00000

467.00000 1894.00000

428.00000 1490.00000

IMAGE=Lioplacis elliptica.tif

ID=17

SCALE=0.002119

LM=36

311.00000 2141.00000

483.00000 2089.00000

1032.00000 2006.00000

1225.00000 1954.00000

1614.00000 1972.00000

763.00000 1934.00000

2121.00000 1841.00000

2332.00000 1813.00000

2342.00000 1751.00000

2115.00000 1661.00000

2042.00000 1692.00000

1932.00000 1585.00000

2494.00000 1906.00000

2459.00000 1823.00000

2521.00000 1630.00000

2380.00000 1713.00000

3852.00000 1740.00000

4111.00000 1475.00000

3618.00000 975.00000

2356.00000 1178.00000

2201.00000 1406.00000

1814.00000 1461.00000

580.00000 1965.00000

739.00000 1896.00000

2477.00000 754.00000

583.00000 1875.00000

1063.00000 1182.00000

1114.00000 1199.00000

1097.00000 937.00000

1304.00000 961.00000

1377.00000 799.00000

1445.00000 1368.00000

1611.00000 1134.00000

1980.00000 668.00000

473.00000 1799.00000

363.00000 1347.00000

IMAGE=Machomena lineata.tif

ID=18

SCALE=0.002123

LM=36

183.00000 2237.00000

359.00000 2151.00000

832.00000 2089.00000

1366.00000 2078.00000

1532.00000 2137.00000

656.00000 2034.00000

1946.00000 2013.00000

2180.00000 2023.00000

2156.00000 1954.00000

1925.00000 1872.00000

1925.00000 1872.00000

1922.00000 1863.00000

2367.00000 2101.00000

2334.00000 2001.00000

2330.00000 1795.00000

2210.00000 1867.00000

4002.00000 1888.00000

4094.00000 1793.00000

3576.00000 1133.00000

2180.00000 1389.00000

1893.00000 1671.00000

1894.00000 1674.00000

483.00000 2047.00000

630.00000 1985.00000

2192.00000 1088.00000

472.00000 1933.00000

1045.00000 1264.00000

1050.00000 1267.00000

1325.00000 878.00000

1325.00000 878.00000

1325.00000 878.00000

1325.00000 878.00000

1325.00000 878.00000

1325.00000 878.00000

350.00000 1847.00000

355.00000 1661.00000

IMAGE=Mesoplatys ochroptera.tif

ID=19

SCALE=0.001948

LM=36

262.00000 2231.00000

402.00000 2182.00000

988.00000 2060.00000

1226.00000 2013.00000

1547.00000 2083.00000

690.00000 2013.00000

2278.00000 1930.00000

2553.00000 1910.00000

2542.00000 1845.00000

2291.00000 1752.00000

2154.00000 1772.00000

1970.00000 1697.00000

2695.00000 1974.00000

2656.00000 1917.00000

2755.00000 1679.00000

2594.00000 1803.00000

3781.00000 1829.00000

3936.00000 1749.00000

3796.00000 1192.00000

2581.00000 1254.00000

2340.00000 1526.00000

1809.00000 1573.00000

521.00000 2060.00000

692.00000 1943.00000

2680.00000 928.00000

545.00000 1941.00000

1027.00000 1340.00000

1091.00000 1350.00000

1270.00000 839.00000

1394.00000 972.00000

1550.00000 697.00000

1467.00000 1355.00000

1664.00000 1106.00000

2167.00000 676.00000

434.00000 1886.00000

361.00000 1386.00000

IMAGE=Microtheca bolinana.tif

ID=20

SCALE=0.001442

LM=36

343.00000 2070.00000

488.00000 2024.00000

993.00000 1969.00000

1200.00000 1941.00000

1387.00000 1998.00000

747.00000 1917.00000

1716.00000 1889.00000

1920.00000 1912.00000

1944.00000 1863.00000

1716.00000 1741.00000

1677.00000 1746.00000

1550.00000 1671.00000

2063.00000 1995.00000

2037.00000 1881.00000

2066.00000 1731.00000

1977.00000 1798.00000

3688.00000 1941.00000

4203.00000 1531.00000

3296.00000 1008.00000

1949.00000 1301.00000

1770.00000 1508.00000

1407.00000 1573.00000

583.00000 1935.00000

723.00000 1866.00000

2011.00000 982.00000

578.00000 1871.00000

915.00000 1345.00000

915.00000 1345.00000

964.00000 1109.00000

1065.00000 1101.00000

1161.00000 907.00000

1244.00000 1381.00000

1299.00000 1259.00000

1584.00000 881.00000

495.00000 1806.00000

462.00000 1531.00000

IMAGE=Novacastria nothofagii.tif

ID=21

SCALE=0.001378

LM=36

498.00000 2272.00000

692.00000 2130.00000

1231.00000 2042.00000

1483.00000 1987.00000

1633.00000 2029.00000

957.00000 1982.00000

2154.00000 1904.00000

2363.00000 1885.00000

2353.00000 1803.00000

2128.00000 1690.00000

2091.00000 1712.00000

2003.00000 1612.00000

2550.00000 1977.00000

2478.00000 1889.00000

2602.00000 1619.00000

2408.00000 1788.00000

3835.00000 1746.00000

4050.00000 1588.00000

3682.00000 966.00000

2418.00000 1194.00000

2239.00000 1461.00000

1910.00000 1492.00000

765.00000 2026.00000

931.00000 1925.00000

2576.00000 707.00000

796.00000 1897.00000

1169.00000 1176.00000

1169.00000 1176.00000

1439.00000 762.00000

1348.00000 917.00000

1446.00000 762.00000

1537.00000 1303.00000

1651.00000 1047.00000

1933.00000 666.00000

643.00000 1871.00000

508.00000 1534.00000

IMAGE=Oidosoma ornatum.tif

ID=22

SCALE=0.002494

LM=36

384.00000 1946.00000

532.00000 1886.00000

1034.00000 1858.00000

1262.00000 1824.00000

1755.00000 1902.00000

814.00000 1790.00000

2516.00000 1775.00000

2827.00000 1796.00000

2840.00000 1718.00000

2612.00000 1632.00000

2485.00000 1658.00000

2340.00000 1593.00000

2952.00000 1871.00000

2944.00000 1772.00000

2996.00000 1614.00000

2845.00000 1689.00000

3965.00000 1695.00000

4136.00000 1573.00000

3897.00000 1275.00000

2985.00000 1223.00000

2708.00000 1397.00000

2296.00000 1425.00000

640.00000 1809.00000

775.00000 1733.00000

3237.00000 964.00000

664.00000 1684.00000

1247.00000 925.00000

1325.00000 969.00000

1255.00000 741.00000

1703.00000 588.00000

1703.00000 588.00000

1835.00000 1181.00000

2630.00000 580.00000

2630.00000 580.00000

495.00000 1627.00000

412.00000 1295.00000

IMAGE=Peltoschema nigrocouspasa.tif

ID=23

SCALE=0.002126

LM=36

232.00000 1938.00000

433.00000 1825.00000

900.00000 1847.00000

1339.00000 1860.00000

1653.00000 1951.00000

700.00000 1738.00000

2048.00000 1763.00000

2289.00000 1819.00000

2267.00000 1713.00000

2029.00000 1600.00000

2029.00000 1600.00000

2029.00000 1600.00000

2552.00000 1904.00000

2436.00000 1775.00000

2496.00000 1518.00000

2299.00000 1622.00000

3794.00000 1775.00000

4045.00000 1553.00000

3731.00000 919.00000

2317.00000 1092.00000

2035.00000 1374.00000

2007.00000 1377.00000

521.00000 1760.00000

675.00000 1688.00000

2393.00000 725.00000

530.00000 1612.00000

1201.00000 872.00000

1201.00000 872.00000

1399.00000 534.00000

1399.00000 531.00000

1399.00000 531.00000

1399.00000 531.00000

1399.00000 531.00000

1399.00000 531.00000

405.00000 1487.00000

355.00000 1274.00000

IMAGE=Phaedonia circumeinctairrpoeita.tif

ID=24

SCALE=0.001554

LM=36

204.00000 2004.00000

392.00000 1904.00000

982.00000 1923.00000

1148.00000 1866.00000

1756.00000 2004.00000

675.00000 1800.00000

2364.00000 1951.00000

2609.00000 1982.00000

2609.00000 1888.00000

2317.00000 1719.00000

2317.00000 1719.00000

2245.00000 1607.00000

2803.00000 2067.00000

2694.00000 1970.00000

2866.00000 1709.00000

2656.00000 1857.00000

3835.00000 1860.00000

4032.00000 1741.00000

3866.00000 1214.00000

2841.00000 1164.00000

2499.00000 1509.00000

1982.00000 1402.00000

502.00000 1785.00000

681.00000 1731.00000

3007.00000 888.00000

552.00000 1681.00000

1167.00000 1060.00000

1255.00000 1092.00000

1364.00000 706.00000

1665.00000 766.00000

1772.00000 581.00000

1524.00000 1233.00000

2267.00000 628.00000

2267.00000 628.00000

439.00000 1609.00000

455.00000 1245.00000

IMAGE=Phyllocharis undulata.tif

ID=25

SCALE=0.002475

LM=36

439.00000 2261.00000

621.00000 2130.00000

1189.00000 2079.00000

1302.00000 2042.00000

1496.00000 2061.00000

866.00000 2026.00000

1969.00000 1951.00000

2145.00000 1957.00000

2157.00000 1879.00000

1988.00000 1778.00000

1891.00000 1791.00000

1762.00000 1672.00000

2342.00000 2054.00000

2295.00000 1963.00000

2364.00000 1760.00000

2226.00000 1860.00000

3760.00000 1992.00000

4139.00000 1634.00000

3590.00000 1318.00000

2233.00000 1340.00000

2063.00000 1553.00000

1722.00000 1612.00000

687.00000 2073.00000

828.00000 1976.00000

2389.00000 901.00000

684.00000 1954.00000

1073.00000 1399.00000

1076.00000 1396.00000

1073.00000 1101.00000

1283.00000 1073.00000

1327.00000 891.00000

1480.00000 1349.00000

1499.00000 1267.00000

2079.00000 760.00000

559.00000 1892.00000

464.00000 1471.00000

IMAGE=Pixis clavigera.tif

ID=26

SCALE=0.002705

LM=36

477.00000 2073.00000

627.00000 1967.00000

1048.00000 1913.00000

1280.00000 1882.00000

1574.00000 1951.00000

809.00000 1863.00000

1872.00000 1800.00000

2161.00000 1788.00000

2129.00000 1747.00000

1866.00000 1672.00000

1781.00000 1669.00000

1697.00000 1644.00000

2327.00000 1885.00000

2283.00000 1804.00000

2317.00000 1637.00000

2189.00000 1713.00000

3932.00000 1659.00000

3935.00000 1666.00000

3596.00000 979.00000

2189.00000 1198.00000

1951.00000 1487.00000

1574.00000 1543.00000

706.00000 1885.00000

797.00000 1791.00000

2261.00000 807.00000

681.00000 1775.00000

994.00000 1158.00000

1041.00000 1217.00000

1063.00000 835.00000

1160.00000 1104.00000

1349.00000 691.00000

1211.00000 1361.00000

1659.00000 691.00000

1659.00000 691.00000

552.00000 1772.00000

352.00000 1415.00000

IMAGE=Plagiomada tarsata.tif

ID=27

SCALE=0.002956

LM=36

248.00000 2017.00000

477.00000 1929.00000

1085.00000 1923.00000

1292.00000 1885.00000

1562.00000 1935.00000

812.00000 1822.00000

2032.00000 1863.00000

2226.00000 1894.00000

2220.00000 1819.00000

2057.00000 1706.00000

1966.00000 1725.00000

1850.00000 1600.00000

2399.00000 1982.00000

2364.00000 1863.00000

2436.00000 1647.00000

2270.00000 1778.00000

3894.00000 1872.00000

4101.00000 1669.00000

3631.00000 1076.00000

2377.00000 1230.00000

2170.00000 1446.00000

1709.00000 1462.00000

609.00000 1851.00000

794.00000 1772.00000

2584.00000 650.00000

596.00000 1735.00000

1051.00000 1101.00000

1145.00000 1114.00000

1164.00000 734.00000

1402.00000 888.00000

1537.00000 593.00000

1544.00000 1192.00000

1678.00000 1007.00000

2117.00000 537.00000

474.00000 1644.00000

408.00000 1239.00000

IMAGE=Planagetes protevs.tif

ID=28

SCALE=0.001927

LM=36

394.00000 2136.00000

566.00000 2075.00000

1070.00000 1990.00000

1352.00000 1950.00000

1484.00000 2002.00000

808.00000 1931.00000

1934.00000 1894.00000

2158.00000 1887.00000

2148.00000 1821.00000

1920.00000 1722.00000

1873.00000 1755.00000

1746.00000 1639.00000

2313.00000 1997.00000

2247.00000 1894.00000

2327.00000 1656.00000

2191.00000 1783.00000

3823.00000 1837.00000

4000.00000 1613.00000

3441.00000 1058.00000

2212.00000 1213.00000

1991.00000 1479.00000

1607.00000 1550.00000

655.00000 1978.00000

787.00000 1891.00000

2254.00000 848.00000

653.00000 1872.00000

980.00000 1310.00000

980.00000 1310.00000

1234.00000 812.00000

1148.00000 1003.00000

1233.00000 806.00000

1341.00000 1325.00000

1440.00000 1135.00000

1819.00000 714.00000

556.00000 1854.00000

295.00000 1427.00000

IMAGE=Platymela sticticollis.tif

ID=29

SCALE=0.002723

LM=36

446.00000 1978.00000

611.00000 1858.00000

1044.00000 1818.00000

1442.00000 1785.00000

1529.00000 1835.00000

855.00000 1750.00000

2200.00000 1731.00000

2426.00000 1752.00000

2431.00000 1705.00000

2254.00000 1585.00000

2174.00000 1592.00000

2087.00000 1517.00000

2556.00000 1821.00000

2528.00000 1741.00000

2608.00000 1538.00000

2466.00000 1649.00000

4063.00000 1439.00000

4124.00000 1354.00000

3651.00000 952.00000

2506.00000 1048.00000

2346.00000 1321.00000

1974.00000 1397.00000

705.00000 1769.00000

846.00000 1691.00000

2591.00000 742.00000

733.00000 1672.00000

1246.00000 1081.00000

1246.00000 1081.00000

1553.00000 582.00000

1458.00000 796.00000

1555.00000 586.00000

1498.00000 1196.00000

1776.00000 893.00000

2035.00000 575.00000

655.00000 1616.00000

361.00000 970.00000

IMAGE=Platyphora pura.tif

ID=30

SCALE=0.003952

LM=36

154.00000 1889.00000

333.00000 1781.00000

848.00000 1755.00000

1341.00000 1750.00000

1694.00000 1844.00000

594.00000 1708.00000

2106.00000 1726.00000

2379.00000 1766.00000

2389.00000 1738.00000

2111.00000 1616.00000

2078.00000 1635.00000

2000.00000 1587.00000

2556.00000 1863.00000

2504.00000 1795.00000

2608.00000 1599.00000

2443.00000 1686.00000

3599.00000 1736.00000

3832.00000 1689.00000

3686.00000 1145.00000

2450.00000 1204.00000

2167.00000 1394.00000

2099.00000 1418.00000

434.00000 1708.00000

580.00000 1656.00000

2490.00000 1022.00000

474.00000 1599.00000

1119.00000 1142.00000

1122.00000 1142.00000

1666.00000 744.00000

1666.00000 744.00000

1666.00000 744.00000

1534.00000 1102.00000

1930.00000 876.00000

1923.00000 876.00000

387.00000 1559.00000

330.00000 1251.00000

IMAGE=Prasocuris phellandrii.tif

ID=31

SCALE=0.001270

LM=36

443.00000 1847.00000

627.00000 1762.00000

1129.00000 1717.00000

1352.00000 1675.00000

1644.00000 1762.00000

865.00000 1637.00000

2021.00000 1627.00000

2287.00000 1644.00000

2306.00000 1585.00000

2061.00000 1472.00000

1993.00000 1467.00000

1927.00000 1399.00000

2448.00000 1719.00000

2410.00000 1637.00000

2523.00000 1458.00000

2356.00000 1559.00000

3887.00000 1590.00000

4101.00000 1420.00000

3632.00000 883.00000

2429.00000 966.00000

2217.00000 1253.00000

1835.00000 1319.00000

726.00000 1686.00000

841.00000 1592.00000

2525.00000 657.00000

709.00000 1590.00000

1155.00000 961.00000

1202.00000 961.00000

1211.00000 657.00000

1381.00000 749.00000

1501.00000 523.00000

1503.00000 1126.00000

1666.00000 907.00000

2024.00000 478.00000

629.00000 1538.00000

481.00000 1154.00000

IMAGE=Procrisina pictipennis.tif

ID=32

SCALE=0.002822

LM=36

323.00000 2207.00000

453.00000 2077.00000

1006.00000 2016.00000

1345.00000 1971.00000

1795.00000 2075.00000

752.00000 1950.00000

2393.00000 1936.00000

2685.00000 1971.00000

2674.00000 1898.00000

2349.00000 1736.00000

2287.00000 1733.00000

2165.00000 1639.00000

2850.00000 2016.00000

2787.00000 1941.00000

2855.00000 1701.00000

2693.00000 1851.00000

3971.00000 1757.00000

4103.00000 1632.00000

3797.00000 1164.00000

2751.00000 1173.00000

2466.00000 1496.00000

2033.00000 1526.00000

545.00000 1985.00000

709.00000 1894.00000

2810.00000 900.00000

549.00000 1884.00000

1117.00000 1168.00000

1117.00000 1168.00000

1221.00000 864.00000

1421.00000 876.00000

1484.00000 662.00000

1369.00000 1408.00000

1725.00000 1105.00000

2259.00000 723.00000

448.00000 1832.00000

328.00000 1265.00000

IMAGE=Aesernia splendens.tif

ID=33

SCALE=0.003703

LM=36

519.00000 2228.00000

686.00000 2167.00000

1147.00000 2115.00000

1381.00000 2066.00000

1675.00000 2122.00000

938.00000 2047.00000

2276.00000 1978.00000

2481.00000 1985.00000

2492.00000 1915.00000

2294.00000 1818.00000

2243.00000 1825.00000

2144.00000 1719.00000

2622.00000 2047.00000

2575.00000 1960.00000

2674.00000 1774.00000

2530.00000 1872.00000

4044.00000 1825.00000

4092.00000 1757.00000

3691.00000 1206.00000

2565.00000 1345.00000

2396.00000 1562.00000

2031.00000 1642.00000

764.00000 2070.00000

936.00000 1995.00000

2676.00000 1001.00000

797.00000 1971.00000

1338.00000 1317.00000

1338.00000 1317.00000

1538.00000 817.00000

1541.00000 820.00000

1541.00000 820.00000

1611.00000 1416.00000

2165.00000 770.00000

2165.00000 770.00000

698.00000 1908.00000

375.00000 1321.00000

IMAGE=Sphaeratrix latifrons.tif

ID=34

SCALE=0.003342

LM=36

305.00000 2109.00000

439.00000 2091.00000

945.00000 2002.00000

1230.00000 1976.00000

1520.00000 2063.00000

707.00000 1964.00000

2021.00000 1917.00000

2271.00000 1953.00000

2297.00000 1887.00000

2050.00000 1759.00000

1984.00000 1759.00000

1863.00000 1686.00000

2443.00000 2035.00000

2391.00000 1943.00000

2452.00000 1741.00000

2339.00000 1837.00000

3856.00000 1910.00000

4122.00000 1712.00000

3583.00000 1168.00000

2372.00000 1258.00000

2184.00000 1550.00000

1786.00000 1623.00000

537.00000 1997.00000

676.00000 1920.00000

2511.00000 949.00000

547.00000 1887.00000

1051.00000 1234.00000

1084.00000 1248.00000

1155.00000 855.00000

1272.00000 1025.00000

1421.00000 775.00000

1376.00000 1399.00000

1574.00000 1201.00000

2024.00000 789.00000

462.00000 1835.00000

502.00000 1333.00000

IMAGE=Sterromela nucea.tif

ID=35

SCALE=0.003306

LM=36

347.00000 2141.00000

542.00000 1995.00000

1067.00000 1941.00000

1644.00000 1901.00000

1644.00000 1901.00000

825.00000 1868.00000

2345.00000 1805.00000

2671.00000 2003.00000

2612.00000 1921.00000

2428.00000 1696.00000

2341.00000 1711.00000

2205.00000 1652.00000

2781.00000 2054.00000

2745.00000 1923.00000

2802.00000 1807.00000

2695.00000 1833.00000

3831.00000 1975.00000

3540.00000 1804.00000

3827.00000 1451.00000

2820.00000 1277.00000

2524.00000 1519.00000

2072.00000 1506.00000

662.00000 1889.00000

813.00000 1832.00000

3029.00000 928.00000

672.00000 1782.00000

1284.00000 1063.00000

1286.00000 1064.00000

1710.00000 572.00000

1470.00000 827.00000

1710.00000 572.00000

1602.00000 1272.00000

1842.00000 1074.00000

2370.00000 634.00000

542.00000 1726.00000

474.00000 1434.00000

IMAGE=Zygogramma sufuralis.tif

ID=36

SCALE=0.001618

LM=36

425.00000 2140.00000

588.00000 2022.00000

1126.00000 1981.00000

1378.00000 1912.00000

1596.00000 1995.00000

843.00000 1905.00000

1976.00000 1881.00000

2280.00000 1874.00000

2280.00000 1822.00000

1958.00000 1705.00000

1903.00000 1708.00000

1793.00000 1632.00000

2442.00000 1967.00000

2414.00000 1881.00000

2428.00000 1663.00000

2324.00000 1781.00000

3757.00000 1843.00000

4003.00000 1622.00000

3540.00000 1045.00000

2362.00000 1218.00000

2152.00000 1480.00000

1706.00000 1549.00000

646.00000 1936.00000

829.00000 1843.00000

2414.00000 900.00000

660.00000 1843.00000

1133.00000 1214.00000

1133.00000 1214.00000

1154.00000 1035.00000

1257.00000 1052.00000

1354.00000 890.00000

1413.00000 1348.00000

1520.00000 1218.00000

1938.00000 796.00000

599.00000 1814.00000

528.00000 1407.00000

IMAGE=Aesernoides nigrofasciata.tif

ID=37

SCALE=0.003838

LM=36

460.00000 2053.00000

591.00000 1960.00000

1109.00000 1908.00000

1282.00000 1850.00000

1568.00000 1939.00000

833.00000 1850.00000

2014.00000 1839.00000

2273.00000 1860.00000

2286.00000 1798.00000

2027.00000 1684.00000

1976.00000 1701.00000

1893.00000 1622.00000

2411.00000 1929.00000

2369.00000 1856.00000

2469.00000 1673.00000

2331.00000 1767.00000

3612.00000 1929.00000

3958.00000 1705.00000

3533.00000 1093.00000

2335.00000 1218.00000

2159.00000 1459.00000

1789.00000 1508.00000

691.00000 1888.00000

829.00000 1794.00000

2400.00000 900.00000

695.00000 1801.00000

1157.00000 1259.00000

1157.00000 1259.00000

1230.00000 1021.00000

1389.00000 969.00000

1478.00000 865.00000

1503.00000 1287.00000

1630.00000 1135.00000

1945.00000 862.00000

581.00000 1756.00000

512.00000 1401.00000

IMAGE=Calomela bartoni.tif

ID=38

SCALE=0.002884

LM=36

453.00000 2043.00000

594.00000 1929.00000

1057.00000 1881.00000

1323.00000 1843.00000

1485.00000 1891.00000

833.00000 1812.00000

1976.00000 1739.00000

2231.00000 1753.00000

2242.00000 1698.00000

1989.00000 1597.00000

1931.00000 1597.00000

1841.00000 1532.00000

2373.00000 1829.00000

2355.00000 1732.00000

2418.00000 1515.00000

2290.00000 1639.00000

3626.00000 1680.00000

4092.00000 1318.00000

3426.00000 848.00000

2238.00000 1083.00000

2103.00000 1335.00000

1713.00000 1435.00000

677.00000 1843.00000

795.00000 1763.00000

2273.00000 793.00000

691.00000 1760.00000

1071.00000 1149.00000

1072.00000 1148.00000

1089.00000 938.00000

1244.00000 892.00000

1282.00000 755.00000

1352.00000 1272.00000

1510.00000 1055.00000

1795.00000 707.00000

598.00000 1738.00000

449.00000 1446.00000

IMAGE=Carystea imperialis.tif

ID=39

SCALE=0.003033

LM=36

325.00000 2171.00000

487.00000 2074.00000

992.00000 2039.00000

1261.00000 1984.00000

1520.00000 2067.00000

722.00000 1957.00000

2086.00000 1950.00000

2276.00000 1960.00000

2293.00000 1888.00000

2027.00000 1749.00000

1979.00000 1756.00000

1931.00000 1694.00000

2466.00000 2039.00000

2418.00000 1953.00000

2504.00000 1711.00000

2338.00000 1843.00000

3654.00000 1943.00000

3972.00000 1777.00000

3606.00000 1193.00000

2383.00000 1228.00000

2076.00000 1528.00000

1803.00000 1542.00000

591.00000 1970.00000

753.00000 1874.00000

2535.00000 883.00000

594.00000 1874.00000

1036.00000 1304.00000

1036.00000 1304.00000

1240.00000 865.00000

1313.00000 1017.00000

1447.00000 800.00000

1427.00000 1401.00000

1627.00000 1114.00000

1989.00000 776.00000

470.00000 1825.00000

449.00000 1428.00000

IMAGE=Chalcolampra sp.tif

ID=40

SCALE=0.001859

LM=36

695.00000 2102.00000

850.00000 1981.00000

1344.00000 1926.00000

1534.00000 1894.00000

1744.00000 1960.00000

1088.00000 1870.00000

2079.00000 1829.00000

2345.00000 1860.00000

2369.00000 1812.00000

2176.00000 1670.00000

2062.00000 1684.00000

2014.00000 1587.00000

2501.00000 1943.00000

2459.00000 1825.00000

2542.00000 1656.00000

2414.00000 1749.00000

3740.00000 1867.00000

4051.00000 1622.00000

3637.00000 1097.00000

2438.00000 1152.00000

2252.00000 1421.00000

1920.00000 1477.00000

919.00000 1894.00000

1061.00000 1808.00000

2539.00000 883.00000

960.00000 1801.00000

1344.00000 1176.00000

1344.00000 1176.00000

1599.00000 751.00000

1489.00000 928.00000

1599.00000 751.00000

1651.00000 1266.00000

1789.00000 1055.00000

2027.00000 714.00000

836.00000 1777.00000

636.00000 1418.00000

IMAGE=Chalcomela sp.tif

ID=41

SCALE=0.003397

LM=36

391.00000 2067.00000

574.00000 1981.00000

1074.00000 1939.00000

1351.00000 1922.00000

1537.00000 1998.00000

805.00000 1870.00000

2031.00000 1853.00000

2259.00000 1870.00000

2290.00000 1825.00000

2041.00000 1701.00000

1958.00000 1698.00000

1879.00000 1649.00000

2428.00000 1957.00000

2404.00000 1867.00000

2459.00000 1684.00000

2331.00000 1774.00000

3695.00000 1870.00000

4079.00000 1677.00000

3578.00000 1138.00000

2345.00000 1197.00000

2186.00000 1477.00000

1803.00000 1560.00000

660.00000 1888.00000

815.00000 1808.00000

2421.00000 903.00000

684.00000 1781.00000

1119.00000 1176.00000

1154.00000 1180.00000

1216.00000 807.00000

1344.00000 941.00000

1423.00000 741.00000

1499.00000 1276.00000

1627.00000 1076.00000

2010.00000 703.00000

577.00000 1736.00000

449.00000 1342.00000

IMAGE=Chrysopartha agricola.tif

ID=42

SCALE=0.003361

LM=36

385.00000 1586.00000

576.00000 1522.00000

1227.00000 1579.00000

1618.00000 1586.00000

2067.00000 1697.00000

743.00000 1458.00000

2642.00000 1628.00000

2769.00000 1621.00000

2793.00000 1590.00000

2729.00000 1541.00000

2666.00000 1560.00000

2590.00000 1513.00000

2835.00000 1675.00000

2835.00000 1675.00000

2868.00000 1614.00000

2876.00000 1621.00000

2876.00000 1623.00000

2876.00000 1623.00000

2876.00000 1623.00000

3359.00000 1489.00000

3361.00000 1489.00000

2743.00000 1498.00000

626.00000 1437.00000

746.00000 1416.00000

3503.00000 1517.00000

666.00000 1371.00000

1189.00000 1029.00000

1399.00000 1046.00000

1192.00000 1031.00000

1399.00000 1050.00000

1399.00000 1050.00000

1399.00000 1050.00000

1399.00000 1050.00000

1399.00000 1050.00000

585.00000 1336.00000

585.00000 1336.00000

IMAGE=Clidonotus gibbosa.tif

ID=43

SCALE=0.002392

LM=36

640.00000 2062.00000

807.00000 1942.00000

1293.00000 1859.00000

1482.00000 1810.00000

1656.00000 1869.00000

1030.00000 1807.00000

1978.00000 1695.00000

2173.00000 1725.00000

2191.00000 1656.00000

2024.00000 1563.00000

1943.00000 1572.00000

1866.00000 1506.00000

2380.00000 1832.00000

2287.00000 1708.00000

2361.00000 1499.00000

2225.00000 1617.00000

3692.00000 1724.00000

3943.00000 1531.00000

3434.00000 895.00000

2253.00000 1034.00000

2066.00000 1303.00000

1767.00000 1371.00000

852.00000 1850.00000

1010.00000 1758.00000

2300.00000 760.00000

857.00000 1722.00000

1175.00000 1083.00000

1175.00000 1083.00000

1144.00000 956.00000

1354.00000 822.00000

1416.00000 673.00000

1451.00000 1192.00000

1567.00000 947.00000

1925.00000 588.00000

767.00000 1713.00000

369.00000 1091.00000

IMAGE=Cyclomela nitida.tif

ID=44

SCALE=0.003438

LM=36

637.00000 2232.00000

798.00000 2126.00000

1298.00000 2062.00000

1496.00000 2036.00000

1654.00000 2114.00000

1048.00000 1998.00000

2031.00000 1998.00000

2284.00000 2015.00000

2291.00000 1947.00000

2085.00000 1838.00000

2031.00000 1845.00000

1934.00000 1746.00000

2432.00000 2107.00000

2397.00000 2008.00000

2446.00000 1850.00000

2328.00000 1918.00000

3800.00000 2024.00000

4036.00000 1843.00000

3567.00000 1286.00000

2442.00000 1340.00000

2210.00000 1605.00000

1781.00000 1642.00000

852.00000 2024.00000

1022.00000 1939.00000

2524.00000 1031.00000

880.00000 1932.00000

1312.00000 1289.00000

1312.00000 1289.00000

1350.00000 1060.00000

1510.00000 1067.00000

1576.00000 958.00000

1550.00000 1470.00000

1729.00000 1166.00000

2026.00000 862.00000

772.00000 1881.00000

670.00000 1647.00000

IMAGE=Diacosma tricolor.tif

ID=45

SCALE=0.002737

LM=36

326.00000 1949.00000

479.00000 1859.00000

937.00000 1829.00000

1175.00000 1779.00000

1326.00000 1852.00000

694.00000 1761.00000

2086.00000 1775.00000

2367.00000 1795.00000

2370.00000 1720.00000

2083.00000 1605.00000

2024.00000 1616.00000

1914.00000 1522.00000

2521.00000 1842.00000

2456.00000 1764.00000

2538.00000 1562.00000

2395.00000 1685.00000

3638.00000 1680.00000

3871.00000 1507.00000

3658.00000 997.00000

2408.00000 1089.00000

2102.00000 1386.00000

1715.00000 1404.00000

545.00000 1765.00000

687.00000 1704.00000

2470.00000 765.00000

569.00000 1678.00000

1041.00000 1164.00000

1041.00000 1164.00000

1253.00000 725.00000

1309.00000 906.00000

1519.00000 586.00000

1319.00000 1258.00000

1545.00000 1013.00000

1996.00000 619.00000

453.00000 1640.00000

437.00000 1211.00000

IMAGE=Eulina curtisi.tif

ID=46

SCALE=0.002695

LM=36

413.00000 2199.00000

551.00000 2118.00000

1046.00000 2085.00000

1282.00000 2046.00000

1448.00000 2126.00000

805.00000 2004.00000

1897.00000 1978.00000

2185.00000 1991.00000

2198.00000 1947.00000

1957.00000 1846.00000

1907.00000 1843.00000

1783.00000 1768.00000

2356.00000 2074.00000

2315.00000 1978.00000

2380.00000 1784.00000

2239.00000 1898.00000

3736.00000 1952.00000

4193.00000 1592.00000

3490.00000 1140.00000

2258.00000 1361.00000

2073.00000 1594.00000

1682.00000 1670.00000

644.00000 2012.00000

797.00000 1958.00000

2294.00000 1044.00000

641.00000 1952.00000

1041.00000 1361.00000

1075.00000 1379.00000

1075.00000 1120.00000

1227.00000 1179.00000

1363.00000 967.00000

1370.00000 1483.00000

1505.00000 1296.00000

1780.00000 938.00000

584.00000 1906.00000

400.00000 1488.00000

IMAGE=Faex orphana.tif

ID=47

SCALE=0.001705

LM=36

457.00000 1929.00000

597.00000 1856.00000

1199.00000 1841.00000

1601.00000 1825.00000

1801.00000 1898.00000

883.00000 1760.00000

2447.00000 1812.00000

2696.00000 1849.00000

2714.00000 1755.00000

2385.00000 1628.00000

2359.00000 1646.00000

2276.00000 1556.00000

2875.00000 1914.00000

2818.00000 1792.00000

2859.00000 1620.00000

2740.00000 1719.00000

3830.00000 1758.00000

4014.00000 1677.00000

3767.00000 1216.00000

2810.00000 1135.00000

2483.00000 1444.00000

2151.00000 1452.00000

709.00000 1784.00000

857.00000 1709.00000

2919.00000 834.00000

732.00000 1688.00000

1321.00000 1076.00000

1321.00000 1076.00000

1425.00000 829.00000

1604.00000 808.00000

1827.00000 603.00000

1544.00000 1283.00000

1884.00000 990.00000

2400.00000 609.00000

626.00000 1651.00000

585.00000 1084.00000

IMAGE=Lamprolina impressicollis.tif

ID=48

SCALE=0.002559

LM=36

411.00000 2167.00000

570.00000 2060.00000

1112.00000 2005.00000

1344.00000 1953.00000

1658.00000 2029.00000

847.00000 1929.00000

2103.00000 1919.00000

2342.00000 1898.00000

2369.00000 1846.00000

2141.00000 1722.00000

2031.00000 1732.00000

1927.00000 1642.00000

2507.00000 1964.00000

2452.00000 1874.00000

2542.00000 1694.00000

2397.00000 1808.00000

3681.00000 1839.00000

3999.00000 1608.00000

3554.00000 1107.00000

2431.00000 1207.00000

2235.00000 1484.00000

1806.00000 1546.00000

681.00000 1967.00000

829.00000 1881.00000

2507.00000 817.00000

684.00000 1860.00000

1095.00000 1231.00000

1095.00000 1231.00000

1202.00000 907.00000

1337.00000 948.00000

1413.00000 783.00000

1385.00000 1380.00000

1575.00000 1104.00000

2079.00000 662.00000

581.00000 1843.00000

474.00000 1411.00000

IMAGE=Macelola geniculata.tif

ID=49

SCALE=0.002559

LM=36

367.00000 2005.00000

518.00000 1929.00000

1088.00000 1870.00000

1285.00000 1791.00000

1465.00000 1843.00000

798.00000 1787.00000

1879.00000 1701.00000

2159.00000 1736.00000

2183.00000 1667.00000

1951.00000 1563.00000

1872.00000 1591.00000

1724.00000 1508.00000

2355.00000 1822.00000

2269.00000 1711.00000

2352.00000 1549.00000

2221.00000 1622.00000

3733.00000 1680.00000

4179.00000 1259.00000

3516.00000 814.00000

2193.00000 1090.00000

1993.00000 1332.00000

1617.00000 1390.00000

626.00000 1808.00000

767.00000 1732.00000

2366.00000 755.00000

619.00000 1715.00000

1033.00000 1100.00000

1033.00000 1100.00000

1050.00000 945.00000

1251.00000 855.00000

1461.00000 610.00000

1330.00000 1187.00000

1475.00000 959.00000

1876.00000 555.00000

518.00000 1701.00000

442.00000 1428.00000

IMAGE=Oomela sp.tif

ID=50

SCALE=0.001219

LM=36

581.00000 2039.00000

729.00000 1967.00000

1199.00000 1877.00000

1406.00000 1815.00000

1606.00000 1888.00000

971.00000 1805.00000

1862.00000 1767.00000

2097.00000 1791.00000

2124.00000 1711.00000

1876.00000 1584.00000

1796.00000 1591.00000

1713.00000 1518.00000

2245.00000 1870.00000

2217.00000 1770.00000

2311.00000 1587.00000

2159.00000 1677.00000

3699.00000 1763.00000

3975.00000 1587.00000

3478.00000 931.00000

2145.00000 1145.00000

1993.00000 1387.00000

1668.00000 1446.00000

809.00000 1836.00000

960.00000 1777.00000

2210.00000 827.00000

795.00000 1767.00000

1095.00000 1169.00000

1095.00000 1169.00000

1109.00000 921.00000

1261.00000 1004.00000

1320.00000 779.00000

1451.00000 1235.00000

1516.00000 1073.00000

1713.00000 714.00000

708.00000 1746.00000

501.00000 1452.00000

IMAGE=Paropsimorpha caudate.tif

ID=51

SCALE=0.002270

LM=36

415.00000 1915.00000

605.00000 1791.00000

1161.00000 1725.00000

1451.00000 1698.00000

1720.00000 1760.00000

898.00000 1656.00000

2110.00000 1615.00000

2387.00000 1639.00000

2400.00000 1577.00000

2141.00000 1459.00000

2079.00000 1452.00000

2017.00000 1408.00000

2545.00000 1718.00000

2487.00000 1629.00000

2594.00000 1459.00000

2438.00000 1532.00000

3920.00000 1504.00000

4144.00000 1339.00000

3664.00000 862.00000

2490.00000 972.00000

2293.00000 1252.00000

1913.00000 1318.00000

688.00000 1708.00000

888.00000 1604.00000

2532.00000 714.00000

729.00000 1604.00000

1175.00000 914.00000

1261.00000 879.00000

1285.00000 572.00000

1465.00000 689.00000

1623.00000 486.00000

1506.00000 1093.00000

1720.00000 893.00000

2169.00000 486.00000

605.00000 1573.00000

480.00000 1135.00000

IMAGE=Paropsis atomaria.tif

ID=52

SCALE=0.004370

LM=36

480.00000 2102.00000

660.00000 2008.00000

1071.00000 1926.00000

1271.00000 1870.00000

1478.00000 1939.00000

867.00000 1863.00000

1910.00000 1784.00000

2166.00000 1808.00000

2166.00000 1736.00000

1948.00000 1660.00000

1893.00000 1670.00000

1789.00000 1563.00000

2311.00000 1874.00000

2286.00000 1787.00000

2324.00000 1597.00000

2231.00000 1694.00000

3830.00000 1711.00000

4075.00000 1463.00000

3436.00000 1021.00000

2207.00000 1155.00000

2079.00000 1414.00000

1720.00000 1501.00000

700.00000 1886.00000

857.00000 1803.00000

2263.00000 886.00000

696.00000 1818.00000

1069.00000 1130.00000

1069.00000 1130.00000

1064.00000 878.00000

1178.00000 982.00000

1280.00000 762.00000

1269.00000 1345.00000

1456.00000 1091.00000

1842.00000 676.00000

587.00000 1753.00000

325.00000 1379.00000

IMAGE=Paropsisterna variabilis.tif

ID=53

SCALE=0.004389

LM=36

277.00000 2135.00000

443.00000 2016.00000

1002.00000 1981.00000

1416.00000 1953.00000

1554.00000 2012.00000

726.00000 1890.00000

2138.00000 1856.00000

2408.00000 1874.00000

2418.00000 1824.00000

2204.00000 1727.00000

2116.00000 1764.00000

1994.00000 1705.00000

2581.00000 1984.00000

2540.00000 1887.00000

2587.00000 1683.00000

2468.00000 1783.00000

3789.00000 1899.00000

4094.00000 1579.00000

3582.00000 1112.00000

2465.00000 1294.00000

2317.00000 1532.00000

1818.00000 1579.00000

547.00000 1921.00000

723.00000 1840.00000

2547.00000 911.00000

547.00000 1837.00000

1027.00000 1278.00000

1090.00000 1306.00000

1137.00000 1017.00000

1272.00000 1140.00000

1432.00000 854.00000

1460.00000 1407.00000

1645.00000 1221.00000

2148.00000 754.00000

449.00000 1761.00000

421.00000 1457.00000

IMAGE=Peltoshema turbata.tif

ID=54

SCALE=0.002001

LM=36

381.00000 2138.00000

562.00000 2082.00000

1077.00000 2034.00000

1413.00000 2028.00000

1586.00000 2113.00000

854.00000 1950.00000

2060.00000 2003.00000

2374.00000 2019.00000

2380.00000 1947.00000

2129.00000 1837.00000

2060.00000 1837.00000

1906.00000 1724.00000

2559.00000 2138.00000

2502.00000 2016.00000

2543.00000 1796.00000

2421.00000 1909.00000

3815.00000 2060.00000

4150.00000 1764.00000

3614.00000 1225.00000

2502.00000 1331.00000

2276.00000 1611.00000

1827.00000 1673.00000

660.00000 1959.00000

836.00000 1887.00000

2584.00000 971.00000

691.00000 1871.00000

1147.00000 1266.00000

1182.00000 1281.00000

1238.00000 895.00000

1423.00000 1048.00000

1548.00000 820.00000

1392.00000 1512.00000

1646.00000 1221.00000

2112.00000 804.00000

593.00000 1790.00000

527.00000 1426.00000

IMAGE=Philhydronopa aneipennis.tif

ID=55

SCALE=0.001756

LM=36

352.00000 2169.00000

534.00000 2060.00000

1109.00000 2006.00000

1360.00000 1965.00000

1671.00000 2050.00000

817.00000 1918.00000

2214.00000 1943.00000

2449.00000 1940.00000

2452.00000 1874.00000

2217.00000 1761.00000

2129.00000 1777.00000

2028.00000 1708.00000

2606.00000 2041.00000

2556.00000 1921.00000

2663.00000 1702.00000

2502.00000 1843.00000

3824.00000 1903.00000

4063.00000 1664.00000

3626.00000 1086.00000

2490.00000 1247.00000

2330.00000 1495.00000

1893.00000 1573.00000

644.00000 1972.00000

810.00000 1881.00000

2650.00000 851.00000

672.00000 1852.00000

1134.00000 1206.00000

1162.00000 1215.00000

1165.00000 860.00000

1394.00000 926.00000

1536.00000 660.00000

1507.00000 1306.00000

1652.00000 1108.00000

2220.00000 660.00000

575.00000 1790.00000

446.00000 1397.00000

IMAGE=Starycea jansoni.tif

ID=56

SCALE=0.001656

LM=36

456.00000 2185.00000

650.00000 2038.00000

1250.00000 1981.00000

1423.00000 1925.00000

1664.00000 2025.00000

933.00000 1906.00000

2063.00000 1887.00000

2352.00000 1918.00000

2355.00000 1837.00000

2101.00000 1695.00000

2032.00000 1695.00000

1959.00000 1617.00000

2512.00000 2034.00000

2449.00000 1915.00000

2559.00000 1686.00000

2396.00000 1802.00000

3749.00000 1972.00000

4025.00000 1830.00000

3654.00000 1099.00000

2424.00000 1212.00000

2270.00000 1479.00000

1878.00000 1523.00000

748.00000 1943.00000

901.00000 1840.00000

2546.00000 813.00000

773.00000 1815.00000

1200.00000 1159.00000

1240.00000 1171.00000

1297.00000 826.00000

1520.00000 889.00000

1620.00000 703.00000

1536.00000 1290.00000

1743.00000 1052.00000

2097.00000 694.00000

679.00000 1771.00000

368.00000 986.00000

IMAGE=Stethomela fulvicollis.tif

ID=57

SCALE=0.003948

LM=36

509.00000 2104.00000

653.00000 2016.00000

1087.00000 1947.00000

1200.00000 1909.00000

1429.00000 1956.00000

839.00000 1909.00000

1749.00000 1837.00000

1997.00000 1871.00000

2003.00000 1786.00000

1740.00000 1677.00000

1683.00000 1692.00000

1620.00000 1636.00000

2160.00000 1978.00000

2082.00000 1846.00000

2170.00000 1630.00000

2032.00000 1755.00000

3771.00000 1790.00000

3934.00000 1507.00000

3212.00000 980.00000

1966.00000 1243.00000

1768.00000 1507.00000

1510.00000 1560.00000

675.00000 1940.00000

820.00000 1862.00000

2050.00000 914.00000

697.00000 1843.00000

1005.00000 1322.00000

1005.00000 1322.00000

1030.00000 1083.00000

1118.00000 1152.00000

1206.00000 923.00000

1269.00000 1419.00000

1369.00000 1215.00000

1667.00000 860.00000

606.00000 1821.00000

468.00000 1529.00000

IMAGE=Tinosis sp.tif

ID=58

SCALE=0.002536

LM=36

362.00000 2078.00000

522.00000 1987.00000

1033.00000 1943.00000

1419.00000 1909.00000

1539.00000 1987.00000

798.00000 1862.00000

2000.00000 1824.00000

2274.00000 1865.00000

2292.00000 1771.00000

2035.00000 1667.00000

1978.00000 1683.00000

1890.00000 1623.00000

2405.00000 1925.00000

2415.00000 1849.00000

2465.00000 1667.00000

2342.00000 1746.00000

3752.00000 1796.00000

4085.00000 1570.00000

3573.00000 1058.00000

2361.00000 1193.00000

2188.00000 1473.00000

1796.00000 1507.00000

644.00000 1893.00000

766.00000 1808.00000

2443.00000 889.00000

644.00000 1793.00000

1127.00000 1155.00000

1128.00000 1164.00000

1141.00000 942.00000

1278.00000 957.00000

1388.00000 732.00000

1344.00000 1409.00000

1568.00000 1117.00000

2032.00000 688.00000

550.00000 1752.00000

468.00000 1297.00000

IMAGE=Trachymela regularis.tif

ID=59

SCALE=0.003809

LM=36

427.00000 2012.00000

594.00000 1896.00000

1159.00000 1847.00000

1347.00000 1812.00000

1617.00000 1887.00000

861.00000 1755.00000

2006.00000 1730.00000

2320.00000 1780.00000

2320.00000 1714.00000

2066.00000 1623.00000

2016.00000 1614.00000

1871.00000 1491.00000

2506.00000 1896.00000

2436.00000 1761.00000

2487.00000 1579.00000

2377.00000 1667.00000

3855.00000 1721.00000

4041.00000 1579.00000

3617.00000 1011.00000

2424.00000 1134.00000

2226.00000 1363.00000

1793.00000 1469.00000

669.00000 1790.00000

839.00000 1705.00000

2531.00000 782.00000

694.00000 1692.00000

971.00000 1243.00000

1109.00000 1137.00000

1194.00000 681.00000

1290.00000 917.00000

1427.00000 632.00000

1321.00000 1314.00000

1534.00000 1104.00000

2169.00000 590.00000

592.00000 1626.00000

364.00000 933.00000

IMAGE=Trochalodes circa.tif

ID=60

SCALE=0.004411

LM=36

297.00000 2361.00000

490.00000 2320.00000

994.00000 2279.00000

1245.00000 2254.00000

1387.00000 2320.00000

680.00000 2230.00000

1904.00000 2151.00000

2169.00000 2168.00000

2173.00000 2116.00000

1845.00000 2006.00000

1845.00000 2006.00000

1669.00000 1965.00000

2414.00000 2268.00000

2311.00000 2175.00000

2325.00000 1985.00000

2235.00000 2061.00000

3966.00000 1958.00000

3966.00000 1958.00000

3645.00000 1399.00000

2201.00000 1554.00000

2232.00000 1692.00000

1966.00000 1820.00000

556.00000 2227.00000

666.00000 2161.00000

2335.00000 1199.00000

559.00000 2127.00000

1028.00000 1410.00000

1025.00000 1499.00000

1025.00000 1361.00000

1401.00000 1027.00000

1401.00000 1027.00000

1297.00000 1623.00000

1663.00000 1072.00000

1663.00000 1072.00000

432.00000 2082.00000

263.00000 1737.00000

IMAGE=Agasta formosa.tif

ID=61

SCALE=0.003729

LM=36

273.00000 1665.00000

432.00000 1585.00000

949.00000 1544.00000

1145.00000 1506.00000

1628.00000 1616.00000

735.00000 1479.00000

2439.00000 1522.00000

2735.00000 1505.00000

2728.00000 1446.00000

2465.00000 1310.00000

2397.00000 1323.00000

2294.00000 1255.00000

2869.00000 1570.00000

2815.00000 1471.00000

2883.00000 1220.00000

2753.00000 1385.00000

4005.00000 1262.00000

4014.00000 1263.00000

3918.00000 819.00000

2838.00000 765.00000

2552.00000 1054.00000

2212.00000 1109.00000

566.00000 1496.00000

701.00000 1427.00000

2945.00000 420.00000

576.00000 1399.00000

1214.00000 713.00000

1214.00000 713.00000

1718.00000 116.00000

1490.00000 444.00000

1718.00000 120.00000

1518.00000 861.00000

1756.00000 647.00000

2304.00000 203.00000

473.00000 1313.00000

307.00000 354.00000

IMAGE=Agrosteella punctata.tif

ID=62

SCALE=0.003319

LM=36

214.00000 2230.00000

411.00000 2147.00000

901.00000 2123.00000

1252.00000 2044.00000

1652.00000 2106.00000

638.00000 2061.00000

2638.00000 2003.00000

2876.00000 1992.00000

2876.00000 1910.00000

2576.00000 1792.00000

2576.00000 1792.00000

2387.00000 1716.00000

3052.00000 2051.00000

2987.00000 1968.00000

3076.00000 1737.00000

2928.00000 1868.00000

4032.00000 1861.00000

4094.00000 1792.00000

4028.00000 1372.00000

3004.00000 1241.00000

2711.00000 1516.00000

2263.00000 1554.00000

521.00000 2041.00000

711.00000 1951.00000

3214.00000 851.00000

580.00000 1941.00000

1338.00000 1196.00000

1338.00000 1196.00000

1801.00000 692.00000

1656.00000 975.00000

1807.00000 696.00000

1838.00000 1358.00000

2004.00000 1154.00000

2504.00000 592.00000

407.00000 1837.00000

273.00000 903.00000

IMAGE=Agrosteomela indica.tif

ID=63

SCALE=0.002896

LM=36

363.00000 2244.00000

507.00000 2127.00000

1004.00000 2072.00000

1432.00000 1996.00000

1621.00000 2075.00000

763.00000 2003.00000

2266.00000 1979.00000

2504.00000 1996.00000

2497.00000 1892.00000

2283.00000 1761.00000

2207.00000 1796.00000

2076.00000 1706.00000

2649.00000 2072.00000

2607.00000 1947.00000

2707.00000 1754.00000

2545.00000 1851.00000

3925.00000 1820.00000

3925.00000 1820.00000

3769.00000 1120.00000

2594.00000 1241.00000

2349.00000 1561.00000

1949.00000 1589.00000

580.00000 2030.00000

745.00000 1965.00000

2711.00000 879.00000

594.00000 1941.00000

1114.00000 1358.00000

1114.00000 1358.00000

1538.00000 661.00000

1328.00000 1072.00000

1538.00000 651.00000

1473.00000 1310.00000

1625.00000 1130.00000

2114.00000 596.00000

476.00000 1861.00000

245.00000 1161.00000

IMAGE=Ambrostoma fortunei.tif

ID=64

SCALE=0.003462

LM=36

294.00000 2110.00000

469.00000 2041.00000

928.00000 2027.00000

1376.00000 1968.00000

1645.00000 2065.00000

728.00000 1941.00000

2369.00000 1930.00000

2590.00000 1906.00000

2611.00000 1844.00000

2394.00000 1716.00000

2273.00000 1737.00000

2145.00000 1641.00000

2749.00000 1996.00000

2714.00000 1868.00000

2787.00000 1665.00000

2621.00000 1782.00000

3914.00000 1751.00000

4066.00000 1637.00000

3714.00000 1130.00000

2690.00000 1168.00000

2432.00000 1472.00000

2107.00000 1510.00000

559.00000 1944.00000

707.00000 1896.00000

2807.00000 816.00000

583.00000 1847.00000

1118.00000 1196.00000

1118.00000 1196.00000

1521.00000 616.00000

1349.00000 941.00000

1525.00000 613.00000

1459.00000 1268.00000

1649.00000 1079.00000

2201.00000 575.00000

452.00000 1789.00000

214.00000 1161.00000

IMAGE=Ambrostoma quadriimpressum.tif

ID=65

SCALE=0.002753

LM=36

374.00000 1782.00000

559.00000 1692.00000

1049.00000 1627.00000

1287.00000 1599.00000

1445.00000 1672.00000

818.00000 1547.00000

1825.00000 1541.00000

2042.00000 1592.00000

2045.00000 1537.00000

1835.00000 1423.00000

1787.00000 1437.00000

1697.00000 1389.00000

2190.00000 1727.00000

2149.00000 1585.00000

2235.00000 1413.00000

2073.00000 1496.00000

3669.00000 1682.00000

4066.00000 1420.00000

3473.00000 806.00000

2145.00000 947.00000

1966.00000 1203.00000

1594.00000 1268.00000

621.00000 1599.00000

801.00000 1510.00000

2238.00000 592.00000

638.00000 1523.00000

990.00000 916.00000

990.00000 913.00000

987.00000 641.00000

1164.00000 696.00000

1213.00000 509.00000

1282.00000 1049.00000

1435.00000 892.00000

1798.00000 473.00000

502.00000 1484.00000

403.00000 1232.00000

IMAGE=Asiparopsis pardalis.tif

ID=66

SCALE=0.003301

LM=36

287.00000 1796.00000

449.00000 1675.00000

990.00000 1641.00000

1207.00000 1606.00000

1604.00000 1717.00000

756.00000 1562.00000

2314.00000 1606.00000

2611.00000 1582.00000

2625.00000 1534.00000

2366.00000 1409.00000

2288.00000 1443.00000

2190.00000 1348.00000

2818.00000 1679.00000

2745.00000 1586.00000

2787.00000 1355.00000

2659.00000 1475.00000

4090.00000 1299.00000

4138.00000 1203.00000

3845.00000 703.00000

2728.00000 837.00000

2487.00000 1134.00000

2073.00000 1131.00000

556.00000 1575.00000

735.00000 1510.00000

2838.00000 506.00000

597.00000 1465.00000

1252.00000 851.00000

1252.00000 851.00000

1663.00000 289.00000

1500.00000 524.00000

1666.00000 286.00000

1607.00000 944.00000

1838.00000 744.00000

2252.00000 206.00000

449.00000 1403.00000

425.00000 703.00000

IMAGE=Chrysolina aeruginoda.tif

ID=67

SCALE=0.001866

LM=36

266.00000 2338.00000

556.00000 2358.00000

645.00000 2368.00000

1332.00000 2282.00000

2156.00000 2434.00000

890.00000 2258.00000

3045.00000 2254.00000

3397.00000 2251.00000

3401.00000 2144.00000

3101.00000 2110.00000

3104.00000 2113.00000

2783.00000 2020.00000

3628.00000 2275.00000

3525.00000 2168.00000

3576.00000 1879.00000

3452.00000 2061.00000

3959.00000 2099.00000

3959.00000 2099.00000

3880.00000 1547.00000

3597.00000 1561.00000

3232.00000 1847.00000

2849.00000 1761.00000

645.00000 2282.00000

890.00000 2185.00000

3825.00000 1199.00000

666.00000 2196.00000

1525.00000 1368.00000

1525.00000 1368.00000

2080.00000 547.00000

1887.00000 972.00000

2083.00000 554.00000

2304.00000 1185.00000

2452.00000 1016.00000

3125.00000 575.00000

614.00000 2054.00000

528.00000 1158.00000

IMAGE=Chrysolina aeruginosa.tif

ID=68

SCALE=0.001462

LM=36

76.00000 2244.00000

318.00000 2241.00000

818.00000 2234.00000

1190.00000 2185.00000

1518.00000 2261.00000

621.00000 2137.00000

2394.00000 2144.00000

2769.00000 2147.00000

2769.00000 2054.00000

2414.00000 1910.00000

2296.00000 1978.00000

2190.00000 1865.00000

2956.00000 2223.00000

2859.00000 2096.00000

2956.00000 1854.00000

2797.00000 1972.00000

4025.00000 1985.00000

4025.00000 1985.00000

4094.00000 1334.00000

2869.00000 1258.00000

2518.00000 1572.00000

2028.00000 1661.00000

449.00000 2158.00000

607.00000 2100.00000

3021.00000 823.00000

494.00000 2051.00000

1238.00000 1254.00000

1245.00000 1257.00000

1466.00000 723.00000

1387.00000 1036.00000

1470.00000 716.00000

1522.00000 1458.00000

1867.00000 1095.00000

2279.00000 575.00000

350.00000 1968.00000

379.00000 1476.00000

IMAGE=Chrysolina aurichakea.tif

ID=69

SCALE=0.002151

LM=36

242.00000 1703.00000

435.00000 1613.00000

901.00000 1592.00000

1263.00000 1554.00000

1514.00000 1630.00000

687.00000 1499.00000

1966.00000 1492.00000

2259.00000 1479.00000

2242.00000 1417.00000

1969.00000 1323.00000

1880.00000 1348.00000

1752.00000 1299.00000

2483.00000 1565.00000

2404.00000 1461.00000

2494.00000 1272.00000

2356.00000 1379.00000

3773.00000 1430.00000

3825.00000 1375.00000

3714.00000 648.00000

2314.00000 810.00000

2042.00000 1106.00000

1735.00000 1199.00000

521.00000 1517.00000

683.00000 1427.00000

2383.00000 485.00000

528.00000 1406.00000

1014.00000 682.00000

1056.00000 703.00000

1025.00000 572.00000

1394.00000 282.00000

1394.00000 282.00000

1314.00000 906.00000

1832.00000 268.00000

1832.00000 268.00000

397.00000 1351.00000

314.00000 1030.00000

IMAGE=Chrysomela populi.tif

ID=70

SCALE=0.003447

LM=36

328.00000 1765.00000

521.00000 1685.00000

952.00000 1630.00000

1218.00000 1596.00000

1497.00000 1682.00000

732.00000 1589.00000

2004.00000 1554.00000

2201.00000 1565.00000

2190.00000 1530.00000

1963.00000 1458.00000

1894.00000 1461.00000

1745.00000 1430.00000

2421.00000 1637.00000

2377.00000 1577.00000

2432.00000 1406.00000

2304.00000 1503.00000

4056.00000 1406.00000

4066.00000 1396.00000

3659.00000 775.00000

2287.00000 985.00000

2001.00000 1265.00000

1966.00000 1275.00000

604.00000 1603.00000

721.00000 1506.00000

2397.00000 644.00000

594.00000 1503.00000

1045.00000 923.00000

1045.00000 923.00000

1466.00000 468.00000

1252.00000 730.00000

1469.00000 461.00000

1359.00000 1034.00000

1463.00000 885.00000

1973.00000 420.00000

476.00000 1430.00000

304.00000 1068.00000

IMAGE=Chrysomela salicivorax.tif

ID=71

SCALE=0.002601

LM=36

380.00000 2106.00000

549.00000 2006.00000

1007.00000 1923.00000

1321.00000 1920.00000

1504.00000 1965.00000

769.00000 1869.00000

1935.00000 1875.00000

2225.00000 1885.00000

2221.00000 1875.00000

1925.00000 1733.00000

1865.00000 1739.00000

1739.00000 1711.00000

2397.00000 1944.00000

2362.00000 1863.00000

2440.00000 1678.00000

2293.00000 1812.00000

3925.00000 1795.00000

4060.00000 1518.00000

3722.00000 1064.00000

2232.00000 1200.00000

1959.00000 1540.00000

1619.00000 1614.00000

623.00000 1918.00000

748.00000 1815.00000

2309.00000 896.00000

621.00000 1798.00000

1090.00000 1144.00000

1088.00000 1147.00000

1421.00000 736.00000

1417.00000 738.00000

1415.00000 735.00000

1290.00000 1320.00000

1849.00000 661.00000

1849.00000 661.00000

480.00000 1761.00000

176.00000 1365.00000

IMAGE=Chrysomela vigintipunctata.tif

ID=72

SCALE=0.002447

LM=36

387.00000 2192.00000

521.00000 2096.00000

1069.00000 2044.00000

1359.00000 2013.00000

1656.00000 2068.00000

807.00000 1975.00000

2156.00000 1972.00000

2394.00000 1999.00000

2404.00000 1923.00000

2204.00000 1813.00000

2111.00000 1820.00000

1973.00000 1727.00000

2652.00000 2113.00000

2528.00000 1951.00000

2552.00000 1699.00000

2445.00000 1841.00000

3845.00000 1879.00000

4032.00000 1765.00000

3732.00000 1137.00000

2487.00000 1279.00000

2201.00000 1537.00000

1776.00000 1558.00000

621.00000 1999.00000

769.00000 1937.00000

2625.00000 892.00000

680.00000 1865.00000

1169.00000 1275.00000

1169.00000 1275.00000

1542.00000 765.00000

1459.00000 906.00000

1545.00000 754.00000

1521.00000 1351.00000

1676.00000 1072.00000

2052.00000 734.00000

514.00000 1820.00000

428.00000 1199.00000

IMAGE=Collaphellus bowringii.tif

ID=73

SCALE=0.001993

LM=36

244.00000 2189.00000

397.00000 2113.00000

894.00000 2061.00000

1259.00000 2023.00000

1466.00000 2089.00000

638.00000 2013.00000

1956.00000 1968.00000

2194.00000 2020.00000

2214.00000 1913.00000

1928.00000 1789.00000

1890.00000 1810.00000

1794.00000 1668.00000

2369.00000 2082.00000

2332.00000 1972.00000

2356.00000 1744.00000

2256.00000 1875.00000

3707.00000 1799.00000

3932.00000 1668.00000

3535.00000 1010.00000

2259.00000 1272.00000

2001.00000 1544.00000

1628.00000 1596.00000

504.00000 2027.00000

632.00000 1961.00000

2363.00000 885.00000

525.00000 1923.00000

935.00000 1323.00000

935.00000 1323.00000

1156.00000 868.00000

1225.00000 982.00000

1342.00000 779.00000

1290.00000 1437.00000

1466.00000 1168.00000

1797.00000 748.00000

373.00000 1861.00000

383.00000 1475.00000

IMAGE=Entomoscelis orientalis.tif

ID=74

SCALE=0.001851

LM=36

283.00000 2316.00000

407.00000 2213.00000

887.00000 2103.00000

1290.00000 2089.00000

1518.00000 2165.00000

714.00000 2044.00000

2159.00000 2051.00000

2438.00000 2075.00000

2490.00000 1985.00000

2221.00000 1882.00000

2104.00000 1923.00000

2021.00000 1799.00000

2607.00000 2151.00000

2576.00000 2020.00000

2621.00000 1868.00000

2521.00000 1944.00000

3952.00000 1875.00000

4149.00000 1713.00000

3711.00000 1137.00000

2521.00000 1299.00000

2297.00000 1589.00000

1876.00000 1616.00000

535.00000 2113.00000

704.00000 1999.00000

2663.00000 1006.00000

563.00000 1982.00000

1156.00000 1265.00000

1156.00000 1265.00000

1225.00000 885.00000

1383.00000 1041.00000

1490.00000 779.00000

1397.00000 1420.00000

1597.00000 1182.00000

2156.00000 706.00000

397.00000 1913.00000

369.00000 1337.00000

IMAGE=Entomoscelis pulla.tif

ID=75

SCALE=0.001774

LM=36

219.00000 1608.00000

418.00000 1536.00000

932.00000 1471.00000

1540.00000 1446.00000

1758.00000 1523.00000

664.00000 1403.00000

2223.00000 1403.00000

2529.00000 1426.00000

2531.00000 1394.00000

2268.00000 1317.00000

2219.00000 1337.00000

2037.00000 1274.00000

2720.00000 1510.00000

2675.00000 1456.00000

2767.00000 1206.00000

2585.00000 1341.00000

4071.00000 1307.00000

4071.00000 1307.00000

3925.00000 741.00000

2548.00000 791.00000

2349.00000 1075.00000

2349.00000 1075.00000

542.00000 1412.00000

696.00000 1302.00000

2690.00000 335.00000

553.00000 1292.00000

1069.00000 778.00000

1067.00000 780.00000

1566.00000 249.00000

1219.00000 660.00000

1572.00000 249.00000

1343.00000 853.00000

1429.00000 821.00000

2251.00000 283.00000

420.00000 1236.00000

249.00000 621.00000

IMAGE=Gastrolina depressa.tif

ID=76

SCALE=0.001700

LM=36

276.00000 1868.00000

469.00000 1775.00000

911.00000 1754.00000

1597.00000 1761.00000

1697.00000 1851.00000

721.00000 1685.00000

2235.00000 1692.00000

2563.00000 1713.00000

2511.00000 1654.00000

2283.00000 1589.00000

2235.00000 1616.00000

2069.00000 1565.00000

2769.00000 1792.00000

2704.00000 1689.00000

2749.00000 1513.00000

2587.00000 1603.00000

4042.00000 1592.00000

4042.00000 1592.00000

3932.00000 985.00000

2590.00000 1013.00000

2307.00000 1320.00000

2169.00000 1337.00000

563.00000 1696.00000

718.00000 1592.00000

2628.00000 623.00000

566.00000 1575.00000

1090.00000 1092.00000

1090.00000 1092.00000

1707.00000 527.00000

1194.00000 992.00000

1707.00000 523.00000

1387.00000 1151.00000

1411.00000 1110.00000

2183.00000 596.00000

466.00000 1534.00000

352.00000 947.00000

IMAGE=Gastrolina tonkinea.tif

ID=77

SCALE=0.001548

LM=36

290.00000 1982.00000

428.00000 1913.00000

887.00000 1913.00000

1287.00000 1889.00000

1459.00000 1975.00000

683.00000 1834.00000

1845.00000 1851.00000

2204.00000 1868.00000

2218.00000 1830.00000

1911.00000 1772.00000

1835.00000 1782.00000

1690.00000 1747.00000

2349.00000 1937.00000

2345.00000 1889.00000

2421.00000 1651.00000

2249.00000 1761.00000

3966.00000 1589.00000

3966.00000 1589.00000

3597.00000 972.00000

2232.00000 1216.00000

1973.00000 1485.00000

1607.00000 1565.00000

535.00000 1827.00000

669.00000 1758.00000

2280.00000 868.00000

535.00000 1741.00000

956.00000 1199.00000

983.00000 1216.00000

1511.00000 710.00000

1128.00000 989.00000

1525.00000 710.00000

1245.00000 1296.00000

1832.00000 737.00000

1832.00000 737.00000

428.00000 1682.00000

394.00000 1406.00000

IMAGE=Gastrolinoides japonica.tif

ID=78

SCALE=0.001824

LM=36

225.00000 2027.00000

411.00000 1954.00000

863.00000 1958.00000

1301.00000 1920.00000

1487.00000 1996.00000

673.00000 1865.00000

2018.00000 1879.00000

2207.00000 1920.00000

2207.00000 1882.00000

1987.00000 1792.00000

1987.00000 1792.00000

1904.00000 1737.00000

2404.00000 2020.00000

2369.00000 1934.00000

2378.00000 1752.00000

2269.00000 1844.00000

3814.00000 2006.00000

4059.00000 1837.00000

3704.00000 1268.00000

2266.00000 1303.00000

2014.00000 1530.00000

2014.00000 1530.00000

514.00000 1865.00000

669.00000 1813.00000

2307.00000 1072.00000

535.00000 1779.00000

1066.00000 1154.00000

1066.00000 1154.00000

1563.00000 703.00000

1563.00000 703.00000

1563.00000 703.00000

1338.00000 1327.00000

1825.00000 789.00000

1828.00000 789.00000

428.00000 1675.00000

276.00000 1141.00000

IMAGE=Gastrophysa atrocyanea.tif

ID=79

SCALE=0.001692

LM=36

232.00000 1858.00000

380.00000 1810.00000

835.00000 1810.00000

1221.00000 1775.00000

1411.00000 1865.00000

645.00000 1727.00000

1887.00000 1796.00000

2045.00000 1813.00000

2056.00000 1737.00000

1928.00000 1679.00000

1880.00000 1682.00000

1887.00000 1685.00000

2228.00000 1885.00000

2190.00000 1782.00000

2245.00000 1630.00000

2118.00000 1706.00000

3087.00000 1703.00000

3097.00000 1696.00000

3142.00000 1058.00000

2080.00000 1261.00000

1866.00000 1485.00000

1866.00000 1485.00000

490.00000 1716.00000

638.00000 1685.00000

2145.00000 1013.00000

518.00000 1613.00000

1118.00000 1096.00000

1122.00000 1100.00000

1482.00000 848.00000

1483.00000 841.00000

1480.00000 839.00000

1370.00000 1188.00000

1751.00000 882.00000

1755.00000 877.00000

420.00000 1556.00000

423.00000 1221.00000

IMAGE=Gastrophysa mannerheimi.tif

ID=80

SCALE=0.001243

LM=36

261.00000 2058.00000

455.00000 1979.00000

1041.00000 1935.00000

1245.00000 1885.00000

1524.00000 1970.00000

715.00000 1857.00000

1944.00000 1832.00000

2182.00000 1901.00000

2198.00000 1785.00000

1938.00000 1675.00000

1881.00000 1694.00000

1756.00000 1612.00000

2417.00000 1992.00000

2330.00000 1847.00000

2344.00000 1656.00000

2232.00000 1728.00000

3765.00000 1910.00000

4029.00000 1744.00000

3508.00000 1058.00000

2289.00000 1174.00000

2073.00000 1453.00000

1609.00000 1525.00000

558.00000 1860.00000

690.00000 1804.00000

2458.00000 797.00000

568.00000 1757.00000

1001.00000 1202.00000

1001.00000 1202.00000

1211.00000 800.00000

1164.00000 954.00000

1214.00000 794.00000

1389.00000 1268.00000

1486.00000 1101.00000

1906.00000 697.00000

446.00000 1703.00000

311.00000 1236.00000

IMAGE=Gonioctena tredecimmaculata.tif

ID=81

SCALE=0.002419

LM=36

524.00000 2287.00000

716.00000 2164.00000

1184.00000 2187.00000

1721.00000 2123.00000

2083.00000 2224.00000

1028.00000 2100.00000

2661.00000 2049.00000

2923.00000 2081.00000

2959.00000 1967.00000

2698.00000 1829.00000

2588.00000 1875.00000

2496.00000 1815.00000

3120.00000 2155.00000

3030.00000 2026.00000

3161.00000 1788.00000

2995.00000 1916.00000

4363.00000 1829.00000

4427.00000 1719.00000

4106.00000 1260.00000

3028.00000 1265.00000

2785.00000 1614.00000

2395.00000 1673.00000

849.00000 2063.00000

1010.00000 2017.00000

3134.00000 948.00000

845.00000 1953.00000

1514.00000 1325.00000

1514.00000 1325.00000

1927.00000 705.00000

1776.00000 1026.00000

1923.00000 705.00000

1822.00000 1508.00000

2652.00000 728.00000

2652.00000 728.00000

693.00000 1875.00000

533.00000 990.00000

IMAGE=Humba cyanicollis.tif

ID=82

SCALE=0.003530

LM=36

349.00000 2262.00000

469.00000 2138.00000

1000.00000 2096.00000

1317.00000 2065.00000

1565.00000 2151.00000

728.00000 2034.00000

2010.00000 1982.00000

2351.00000 2000.00000

2334.00000 1941.00000

2051.00000 1851.00000

1962.00000 1879.00000

1803.00000 1810.00000

2516.00000 2072.00000

2468.00000 2000.00000

2531.00000 1781.00000

2358.00000 1893.00000

3950.00000 1910.00000

3950.00000 1910.00000

3774.00000 1262.00000

2379.00000 1345.00000

2092.00000 1645.00000

1920.00000 1669.00000

552.00000 2038.00000

697.00000 1976.00000

2427.00000 1011.00000

576.00000 1931.00000

1014.00000 1235.00000

1052.00000 1283.00000

1072.00000 993.00000

1176.00000 1117.00000

1400.00000 818.00000

1296.00000 1497.00000

1448.00000 1231.00000

1930.00000 804.00000

414.00000 1876.00000

211.00000 1510.00000

IMAGE=Linaeidea aenea.tif

ID=83

SCALE=0.002473

LM=36

311.00000 2065.00000

473.00000 1955.00000

938.00000 1917.00000

1410.00000 1907.00000

1586.00000 1962.00000

700.00000 1865.00000

1989.00000 1855.00000

2313.00000 1920.00000

2306.00000 1851.00000

2048.00000 1741.00000

1965.00000 1769.00000

1827.00000 1679.00000

2527.00000 2003.00000

2447.00000 1924.00000

2487.00000 1694.00000

2369.00000 1812.00000

3933.00000 1927.00000

3933.00000 1927.00000

3819.00000 1297.00000

2447.00000 1304.00000

2089.00000 1579.00000

2089.00000 1579.00000

559.00000 1876.00000

669.00000 1820.00000

2523.00000 935.00000

569.00000 1796.00000

1065.00000 1142.00000

1093.00000 1183.00000

1114.00000 993.00000

1534.00000 690.00000

1534.00000 690.00000

1303.00000 1352.00000

1962.00000 735.00000

1962.00000 735.00000

455.00000 1755.00000

293.00000 1376.00000

IMAGE=Linaeidea placida.tif

ID=84

SCALE=0.002467

LM=36

449.00000 1565.00000

635.00000 1431.00000

1131.00000 1372.00000

1341.00000 1344.00000

1424.00000 1389.00000

859.00000 1282.00000

1924.00000 1272.00000

2144.00000 1317.00000

2161.00000 1269.00000

1975.00000 1113.00000

1893.00000 1138.00000

1834.00000 1044.00000

2320.00000 1417.00000

2245.00000 1311.00000

2344.00000 1089.00000

2196.00000 1214.00000

3457.00000 1269.00000

3805.00000 1069.00000

3412.00000 576.00000

2234.00000 686.00000

2089.00000 948.00000

1696.00000 914.00000

717.00000 1320.00000

845.00000 1255.00000

2382.00000 307.00000

766.00000 1196.00000

1131.00000 686.00000

1131.00000 686.00000

1462.00000 235.00000

1369.00000 377.00000

1462.00000 235.00000

1407.00000 834.00000

1603.00000 472.00000

2010.00000 124.00000

569.00000 1155.00000

545.00000 789.00000

IMAGE=Lycaria westermanni.tif

ID=85

SCALE=0.003906

LM=36

224.00000 2228.00000

404.00000 2093.00000

907.00000 2041.00000

1200.00000 2013.00000

1472.00000 2076.00000

721.00000 1941.00000

2151.00000 1907.00000

2396.00000 1886.00000

2347.00000 1814.00000

2151.00000 1710.00000

2075.00000 1734.00000

2024.00000 1696.00000

2644.00000 2013.00000

2506.00000 1870.00000

2568.00000 1683.00000

2433.00000 1783.00000

3874.00000 1707.00000

4019.00000 1617.00000

3792.00000 1073.00000

2368.00000 1200.00000

2158.00000 1455.00000

2158.00000 1455.00000

552.00000 1986.00000

673.00000 1903.00000

2523.00000 852.00000

552.00000 1855.00000

1041.00000 1093.00000

1041.00000 1093.00000

1451.00000 704.00000

1455.00000 704.00000

1455.00000 704.00000

1338.00000 1290.00000

1851.00000 749.00000

1851.00000 749.00000

390.00000 1772.00000

352.00000 1517.00000

IMAGE=Neophaedon pynitosus aeutacrus.tif

ID=86

SCALE=0.001275

LM=36

207.00000 2200.00000

409.00000 2051.00000

947.00000 2051.00000

1285.00000 2018.00000

1585.00000 2084.00000

701.00000 1959.00000

2098.00000 1943.00000

2411.00000 1968.00000

2423.00000 1897.00000

2111.00000 1842.00000

2111.00000 1842.00000

2111.00000 1847.00000

2607.00000 2064.00000

2607.00000 1955.00000

2582.00000 1742.00000

2465.00000 1830.00000

3841.00000 1792.00000

3854.00000 1784.00000

3741.00000 1217.00000

2453.00000 1255.00000

2173.00000 1521.00000

2173.00000 1521.00000

538.00000 1959.00000

676.00000 1901.00000

2590.00000 896.00000

576.00000 1817.00000

818.00000 992.00000

1122.00000 975.00000

1122.00000 975.00000

1122.00000 975.00000

1122.00000 975.00000

1122.00000 975.00000

1122.00000 975.00000

1122.00000 975.00000

367.00000 1755.00000

338.00000 1521.00000

IMAGE=Odontedon fulvescens.tif

ID=87

SCALE=0.001281

LM=36

1016.00000 2150.00000

1172.00000 2017.00000

1782.00000 1850.00000

1960.00000 1794.00000

2211.00000 1854.00000

1521.00000 1782.00000

2711.00000 1703.00000

2999.00000 1733.00000

3018.00000 1661.00000

2741.00000 1540.00000

2669.00000 1555.00000

2518.00000 1475.00000

3202.00000 1850.00000

3124.00000 1710.00000

3196.00000 1487.00000

3048.00000 1604.00000

4917.00000 1650.00000

5259.00000 1505.00000

4591.00000 736.00000

3048.00000 948.00000

2848.00000 1263.00000

2404.00000 1392.00000

1267.00000 1877.00000

1498.00000 1733.00000

3139.00000 599.00000

1263.00000 1786.00000

1676.00000 1051.00000

1676.00000 1051.00000

1653.00000 717.00000

1832.00000 819.00000

1885.00000 565.00000

1979.00000 1195.00000

2123.00000 1013.00000

2662.00000 417.00000

1043.00000 1706.00000

800.00000 1157.00000

IMAGE=Paropsides nigrosparsa.tif

ID=88

SCALE=0.004374

LM=36

364.00000 2036.00000

524.00000 1991.00000

990.00000 1938.00000

1153.00000 1907.00000

1396.00000 1991.00000

766.00000 1866.00000

1892.00000 1862.00000

2131.00000 1854.00000

2161.00000 1801.00000

1957.00000 1699.00000

1904.00000 1703.00000

1786.00000 1646.00000

2313.00000 1960.00000

2252.00000 1866.00000

2340.00000 1631.00000

2205.00000 1752.00000

3829.00000 1809.00000

4095.00000 1585.00000

3511.00000 1024.00000

2211.00000 1179.00000

2021.00000 1456.00000

1650.00000 1513.00000

603.00000 1888.00000

740.00000 1839.00000

2286.00000 834.00000

603.00000 1813.00000

994.00000 1164.00000

1043.00000 1179.00000

1016.00000 944.00000

1157.00000 975.00000

1263.00000 751.00000

1339.00000 1331.00000

1517.00000 1073.00000

1763.00000 713.00000

497.00000 1767.00000

232.00000 1312.00000

IMAGE=Paropsides soriculata.tif

ID=89

SCALE=0.002516

LM=36

148.00000 1881.00000

327.00000 1741.00000

888.00000 1714.00000

1278.00000 1703.00000

1578.00000 1809.00000

637.00000 1634.00000

1926.00000 1661.00000

2169.00000 1699.00000

2196.00000 1650.00000

1945.00000 1502.00000

1881.00000 1517.00000

1790.00000 1460.00000

2377.00000 1816.00000

2302.00000 1710.00000

2370.00000 1509.00000

2237.00000 1585.00000

3670.00000 1722.00000

3909.00000 1574.00000

3651.00000 956.00000

2245.00000 986.00000

1945.00000 1274.00000

1843.00000 1308.00000

444.00000 1676.00000

622.00000 1566.00000

2374.00000 656.00000

478.00000 1528.00000

979.00000 910.00000

1020.00000 948.00000

1153.00000 603.00000

1426.00000 497.00000

1426.00000 497.00000

1255.00000 1051.00000

1312.00000 948.00000

1900.00000 448.00000

338.00000 1521.00000

338.00000 1229.00000

IMAGE=Phaedon brassicae.tif

ID=90

SCALE=0.001358

LM=36

187.00000 2252.00000

342.00000 2214.00000

846.00000 2150.00000

1066.00000 2116.00000

1335.00000 2188.00000

599.00000 2089.00000

1998.00000 2131.00000

2245.00000 2135.00000

2245.00000 2044.00000

1926.00000 1919.00000

1881.00000 1938.00000

1805.00000 1847.00000

2396.00000 2237.00000

2359.00000 2139.00000

2423.00000 1934.00000

2286.00000 2013.00000

3765.00000 2154.00000

4072.00000 1972.00000

3792.00000 1369.00000

2317.00000 1342.00000

1979.00000 1672.00000

1638.00000 1695.00000

429.00000 2112.00000

569.00000 2032.00000

2434.00000 975.00000

444.00000 2017.00000

861.00000 1468.00000

861.00000 1468.00000

1123.00000 975.00000

1270.00000 1028.00000

1361.00000 869.00000

1214.00000 1555.00000

1494.00000 1221.00000

1858.00000 853.00000

315.00000 1976.00000

270.00000 1562.00000

IMAGE=Phola octodecimguttata.tif

ID=91

SCALE=0.001680

LM=36

238.00000 2134.00000

359.00000 2079.00000

783.00000 2003.00000

1158.00000 1920.00000

1551.00000 2007.00000

604.00000 1938.00000

1862.00000 1876.00000

2061.00000 1900.00000

2003.00000 1831.00000

1831.00000 1796.00000

1827.00000 1796.00000

1727.00000 1731.00000

2237.00000 1958.00000

2148.00000 1876.00000

2192.00000 1714.00000

2052.00000 1784.00000

3850.00000 1734.00000

4195.00000 1379.00000

3492.00000 1000.00000

2044.00000 1300.00000

1927.00000 1434.00000

1779.00000 1541.00000

462.00000 1976.00000

576.00000 1896.00000

2106.00000 1042.00000

442.00000 1886.00000

917.00000 1335.00000

955.00000 1407.00000

1028.00000 1066.00000

1262.00000 900.00000

1262.00000 900.00000

1224.00000 1317.00000

1586.00000 914.00000

1586.00000 914.00000

328.00000 1848.00000

314.00000 1507.00000

IMAGE=Phratora bicolor.tif

ID=92

SCALE=0.002001

LM=36

247.00000 1991.00000

361.00000 1964.00000

842.00000 1900.00000

1062.00000 1888.00000

1316.00000 1960.00000

592.00000 1847.00000

1809.00000 1877.00000

1976.00000 1892.00000

1957.00000 1824.00000

1790.00000 1771.00000

1756.00000 1771.00000

1737.00000 1737.00000

2127.00000 1960.00000

2066.00000 1881.00000

2125.00000 1693.00000

1995.00000 1790.00000

3598.00000 1945.00000

4042.00000 1642.00000

3348.00000 1077.00000

1979.00000 1346.00000

1786.00000 1540.00000

1718.00000 1540.00000

452.00000 1873.00000

573.00000 1786.00000

2074.00000 1028.00000

463.00000 1801.00000

952.00000 1274.00000

967.00000 1342.00000

1047.00000 1047.00000

1369.00000 899.00000

1369.00000 899.00000

1244.00000 1282.00000

1665.00000 944.00000

1665.00000 944.00000

380.00000 1782.00000

406.00000 1350.00000

IMAGE=Phratora laticollis.tif

ID=93

SCALE=0.001786

LM=36

243.00000 1403.00000

402.00000 1366.00000

883.00000 1321.00000

992.00000 1309.00000

1228.00000 1349.00000

630.00000 1281.00000

1718.00000 1264.00000

2020.00000 1269.00000

2020.00000 1235.00000

1732.00000 1161.00000

1658.00000 1175.00000

1541.00000 1155.00000

2194.00000 1352.00000

2157.00000 1294.00000

2225.00000 1093.00000

2063.00000 1189.00000

3914.00000 1284.00000

4102.00000 1084.00000

3615.00000 475.00000

2083.00000 702.00000

1789.00000 984.00000

1789.00000 984.00000

473.00000 1281.00000

616.00000 1209.00000

2185.00000 378.00000

479.00000 1198.00000

952.00000 643.00000

952.00000 643.00000

1342.00000 215.00000

1342.00000 215.00000

1342.00000 215.00000

1188.00000 731.00000

1701.00000 213.00000

1701.00000 213.00000

394.00000 1170.00000

328.00000 868.00000

IMAGE=Plagiodera versicolora.tif

ID=94

SCALE=0.001540

LM=36

133.00000 2192.00000

308.00000 2059.00000

910.00000 2002.00000

1604.00000 2025.00000

1604.00000 2025.00000

596.00000 1945.00000

2252.00000 1896.00000

2559.00000 1960.00000

2563.00000 1904.00000

2218.00000 1733.00000

2188.00000 1744.00000

2101.00000 1691.00000

2749.00000 2029.00000

2667.00000 1934.00000

2720.00000 1750.00000

2590.00000 1860.00000

3757.00000 1813.00000

3905.00000 1706.00000

3757.00000 1350.00000

2688.00000 1172.00000

2393.00000 1445.00000

2116.00000 1445.00000

471.00000 1941.00000

596.00000 1888.00000

2874.00000 933.00000

478.00000 1809.00000

1043.00000 1104.00000

1096.00000 1111.00000

1111.00000 747.00000

1305.00000 797.00000

1396.00000 599.00000

1456.00000 1312.00000

1631.00000 1001.00000

2169.00000 546.00000

323.00000 1756.00000

410.00000 1255.00000

IMAGE=Potaninia assamensis.tif

ID=95

SCALE=0.002273

LM=36

292.00000 2108.00000

467.00000 2051.00000

937.00000 1983.00000

1380.00000 1938.00000

1589.00000 1987.00000

736.00000 1941.00000

2260.00000 1862.00000

2628.00000 1926.00000

2677.00000 1862.00000

2374.00000 1684.00000

2260.00000 1714.00000

2177.00000 1676.00000

2798.00000 1972.00000

2764.00000 1858.00000

2863.00000 1665.00000

2690.00000 1788.00000

4080.00000 1767.00000

4080.00000 1767.00000

3807.00000 1229.00000

2802.00000 1210.00000

2544.00000 1437.00000

2067.00000 1475.00000

577.00000 1945.00000

728.00000 1892.00000

2939.00000 914.00000

629.00000 1850.00000

1236.00000 1168.00000

1270.00000 1187.00000

1407.00000 819.00000

1536.00000 925.00000

1714.00000 668.00000

1460.00000 1392.00000

1877.00000 1115.00000

2468.00000 615.00000

482.00000 1797.00000

311.00000 948.00000

IMAGE=Spherolina balyi.tif

ID=96

SCALE=0.004008
